# Increased Biosynthetic Gene Dosage in a Genome-Reduced Defensive Bacterial Symbiont

**DOI:** 10.1128/mSystems.00096-17

**Published:** 2017-11-21

**Authors:** Juan Lopera, Ian J. Miller, Kerry L. McPhail, Jason C. Kwan

**Affiliations:** a Division of Pharmaceutical Sciences, School of Pharmacy, University of Wisconsin—Madison, Madison, Wisconsin, USA; b Department of Pharmaceutical Sciences, College of Pharmacy, Oregon State University, Corvallis, Oregon, USA; University of British Columbia

**Keywords:** *Verrucomicrobia*, metagenomics, natural products, polyketides, symbiosis

## Abstract

A symbiotic lifestyle frequently results in genome reduction in bacteria; the isolation of small populations promotes genetic drift and the fixation of deletions and deleterious mutations over time. Transitions in lifestyle, including host restriction or adaptation to an intracellular habitat, are thought to precipitate a wave of sequence degradation events and consequent proliferation of pseudogenes. We describe here a verrucomicrobial symbiont of the tunicate *Lissoclinum* sp. that appears to be undergoing such a transition, with low coding density and many identifiable pseudogenes. However, despite the overall drive toward genome reduction, this symbiont maintains seven copies of a large polyketide synthase (PKS) pathway for the mandelalides (*mnd*), cytotoxic compounds that likely constitute a chemical defense for the host. There is evidence of ongoing degradation in a small number of these repeats—including variable borders, internal deletions, and single nucleotide polymorphisms (SNPs). However, the gene dosage of most of the pathway is increased at least 5-fold. Correspondingly, this single pathway accounts for 19% of the genome by length and 25.8% of the coding capacity. This increased gene dosage in the face of generalized sequence degradation and genome reduction suggests that *mnd* genes are under strong purifying selection and are important to the symbiotic relationship.

**IMPORTANCE** Secondary metabolites, which are small-molecule organic compounds produced by living organisms, provide or inspire drugs for many different diseases. These natural products have evolved over millions of years to provide a survival benefit to the producing organism and often display potent biological activity with important therapeutic applications. For instance, defensive compounds in the environment may be cytotoxic to eukaryotic cells, a property exploitable for cancer treatment. Here, we describe the genome of an uncultured symbiotic bacterium that makes such a cytotoxic metabolite. This symbiont is losing genes that do not endow a selective advantage in a hospitable host environment. Secondary metabolism genes, however, are repeated multiple times in the genome, directly demonstrating their selective advantage. This finding shows the strength of selective forces in symbiotic relationships and suggests that uncultured bacteria in such relationships should be targeted for drug discovery efforts.

**Author Video**: An author video summary of this article is available.

## Abstract (precis)

Secondary metabolites, which are small-molecule organic compounds produced by living organisms, provide or inspire drugs for many different diseases. These natural products have evolved over millions of years to provide a survival benefit to the producing organism and often display potent biological activity with important therapeutic applications. For instance, defensive compounds in the environment may be cytotoxic to eukaryotic cells, a property exploitable for cancer treatment. Here, we describe the genome of an uncultured symbiotic bacterium that makes such a cytotoxic metabolite. This symbiont is losing genes that do not endow a selective advantage in a hospitable host environment. Secondary metabolism genes, however, are repeated multiple times in the genome, directly demonstrating their selective advantage. This finding shows the strength of selective forces in symbiotic relationships and suggests that uncultured bacteria in such relationships should be targeted for drug discovery efforts.

## INTRODUCTION

Microbes frequently associate with higher organisms, and under certain circumstances, such a relationship leads to genome erosion in the microbial partner (1, 2). Host restriction, where an organism is an obligate symbiont with no free-living phase in its life cycle (such as in strict vertical transmission), reduces the need to maintain functions required for independent life. Likewise, adaptation to an intracellular lifestyle further reduces the need to synthesize metabolites available from the host. Sequence degradation and genome reduction occur in the absence of selection pressure, often accompanied by a change in symbiont population structure. Isolated populations of symbionts within hosts undergo frequent population bottlenecks at host-cell division or vertical transmission. In this setting, slightly deleterious mutations can easily become fixed, due to these bottlenecks and the unavailability of horizontal gene transfer (HGT) processes (3). Such sequence degradation leads to weakening of protein function until coding sequences (CDSs) become nonfunctional pseudogenes, which tend to be deleted (1). Transitions to a symbiont lifestyle are therefore accompanied by a proliferation of pseudogenes and apparent lowering of coding density (4) before intergenic sequences are deleted, resulting in vastly reduced genomes.

We have a longstanding interest in symbionts that make bioactive natural products (secondary metabolites) and previously identified a tunicate symbiont that was the source of the patellazoles (5, 6), potent cytotoxins that likely act as chemical defenses for the host. The biosynthetic genes for the patellazoles showed an unusual degree of fragmentation, whereas genes in secondary metabolite pathways tend to be clustered (7). As more symbiont genomes have been sequenced, we have noted that fragmentation of biosynthetic gene clusters (BGCs) appears to be common in symbionts (7).

As part of our efforts to discover novel biosynthetic pathways, we focused on the mandelalides (8–10), which are cytotoxic compounds isolated from the marine tunicate *Lissoclinum* sp. Given the propensity of this genus to have both intra- and extracellular symbionts and the resemblance of the mandelalides to bacterial compounds made by *trans*-AT polyketide synthases (PKS), we embarked on a metagenomic sequencing campaign to characterize the *mnd* pathway and the genome of the producing symbiont. Here, we describe a symbiont in the phylum *Verrucomicrobia*, the genome for which contains a complete set of biosynthetic genes that likely produce the mandelalides. This genome shows signs of ongoing degradation, with numerous pseudogenes and low coding density. To our surprise, the mandelalides gene cluster had much higher coverage than the rest of the genome, and we found evidence that it is connected to multiple parts of the otherwise well-assembled genome. The cluster is repeated seven times and is likely under strong selective pressures to enhance mandelalide production. We also found evidence that the *mnd* cluster is not a recent acquisition and that it is undergoing degradation and sequence divergence. The repeat structure may represent a paradigm for the ancestral state of older symbionts with pathways formed from fragmented secondary metabolite genes.

## RESULTS

### Identification of a bacterial symbiont associated with mandelalide-containing *Lissoclinum* sp.

**FIG 1:**
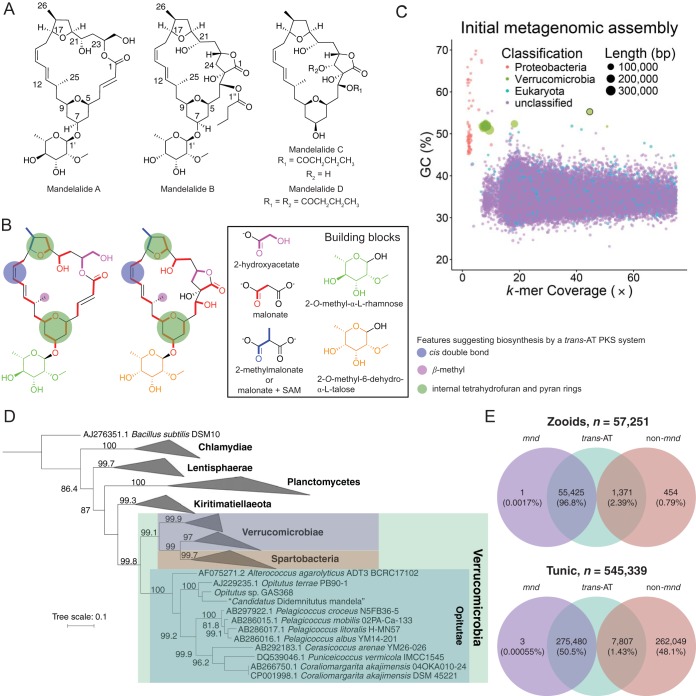
(A) Structures of mandelalides A to D. (B) Retrobiosynthetic analysis of the two types of mandelalide carbon skeleton, showing probable building blocks and features suggestive of *trans*-AT-type polyketide synthases (PKS). (C) Visualization of the metagenomic assembly obtained from the zooid fraction, where each point represents a contig of >3,000 bp in length. Points are colored based on taxonomic group, and their size is proportional to contig length. The contig bearing the *mnd* pathway is outlined in black. (D) Approximately maximum-likelihood tree based on 16S rRNA gene sequences from “*Candidatus* Didemnitutus mandela” and 100 other bacteria in the *Planctomycetes*-*Verrucomicrobia*-*Chlamydiae* superphylum obtained from the Ribosomal Database Project (RDP) (83), showing the placement of “*Ca*. Didemnitutus mandela” in the family *Opitutae* in the phylum *Verrucomicrobia*. Bootstrap proportions greater than 70% are expressed to the left of each node as a percentage of 1,000 replicates. (E) Amplicon analysis of ketosynthase (KS) domains in *Lissoclinum* sp. zooid and tunic fractions. *mnd* accounts for the vast majority of KS domains in the zooid fraction and for *trans*-AT KS domains in both fractions.

In an effort to investigate the biosynthesis of the mandelalides, we recollected the *Lissoclinum* sp. tunicate that had previously yielded the mandelalides (8), near the original collection site of Algoa Bay, South Africa. The individual animal that we collected yielded mandelalides A to D (Fig. 1A), as well as eight new analog mandelalides, E through L (9, 11). Tunicates in the genus *Lissoclinum* are colonial, consisting of many tiny individual animals (zooids) enveloped in a protective coat or “tunic.” The mandelalide-containing tunicate was dissected to separate the tunic from the zooids, since we previously found a bacterial symbiont of the related tunicate *Lissoclinum patella* to be localized to zooids (5). Unlike *L. patella* (12), the mandelalide-containing animal appeared not to harbor *Prochloron didemni* or other photosynthetic symbionts in the cloacal contents. Total DNA was extracted separately from each of the tunic and zooid fractions and subjected to shotgun metagenomic sequencing (Illumina 101-bp paired end). Retrobiosynthetic analysis of mandelalide structures revealed several features suggestive of synthesis via a *trans*-acyltransferase (AT) polyketide synthase (PKS) pathway (13, 14). These included the presence of a *cis* double bond, a β-methyl moiety, and multiple tetrahydrofuran (THF) and -pyran (THP) rings (Fig. 1B). Therefore, we searched initial assemblies for fragments of *trans*-AT pathways. One putative pathway was found; however, it was fragmented due to low coverage and there was a general lack of bacterial contigs in the metagenome. Since coverage of this putative pathway was higher in the zooid fraction than in the tunic, additional sequencing was obtained from the zooid extract.

With the additional sequencing data in hand (Table 1), a new metagenome assembly was constructed from all zooid-derived sequence reads, with tunic-derived reads excluded. Predicted open reading frames (ORFs) within the contigs were used to infer probable taxonomy based on the lowest common ancestor of BLASTP hits in the NCBI nr database (15, 16). In visualizations of the assembly, a discrete cluster in the bacterial phylum *Verrucomicrobia* was observed, comprised of contigs with low coverage and high GC content (Fig. 1C). This set, termed Ver_v1 here, consisted of 15 contigs that we predicted to represent a bacterial genome that was 94.2% complete and 100% pure, based on analysis of single-copy markers (Table 2) (17). This assembly included a large 108-kbp contig containing a complete *trans*-AT PKS pathway that we termed *mnd*. Unexpectedly, this large contig showed much higher coverage than the other contigs in Ver_v1, suggesting either a sequence misassembly or that the *mnd* pathway is repeated within the symbiont genome.

**TABLE 1:** Sequence data used in this study

Method of sequencing and type of value	Tunic	Zooids
Whole metagenome		
Illumina HiSeq reads (2 × 101 bp), millions	96.2	279.1
Assembly, thousands of contigs		2,575
Assembly, Mbp		1,009
Assembly, *N*_50_, bp		2,453
KS PCR, Illumina MiSeq reads (2 × 251 bp), thousands	545.3	57.3
16S PCR, Illumina MiSeq reads (2 × 251 bp), thousands	92.6	0.53

**TABLE 2:** Assembly characteristics

Characteristic	Value for assembly:	
	Ver_v1	Ver_v2
Size (Mbp)	2.17	2.17
No. of contigs	15	10
*N*_50_ (kbp)	224.8	319.6
GC content (%)	51.93	51.93
Completeness (%)	94.2	94.2
Purity (%)	100	100

To obtain greater coverage of the symbiont genome and potentially achieve a better assembly, the subset of reads from both zooid and tunic DNA extracts that aligned to Ver_v1 contigs was segregated and reassembled. The lower-coverage contigs from Ver_v1 did indeed coalesce into fewer contigs, although the higher-coverage contig, including the section containing the *mnd* genes, became fragmented. We hypothesize that in the single-genome setting and with higher coverage, the repeat structure of *mnd* was more apparent to the assembler (see below), resulting in fragmented contigs that could not be resolved automatically. By alignment of paired-end reads, we confirmed that the original *mnd* sequence obtained from Ver_v1 was consistent with most of the repeats. We combined the genomic contigs obtained from the new assembly, along with the original *mnd* contig, in Ver_v2 (Table 2). This genome assembly is 2.17 Mbp in total length and estimated to be 94.2% complete and 100% pure. Phylogenetic analysis of the full-length 16S rRNA gene places the symbiont, which we termed “*Candidatus* Didemnitutus mandela,” in a clade with the family *Opitutaceae* in the phylum *Verrucomicrobia* (Fig. 1D), and this placement is consistent with a phylogenetic tree derived from concatenated protein markers (see Fig. S1 in the supplemental material). In a similar vein as the naming of *Opitutus* (“protected by the Roman Earth and harvest goddess Ops” [18]), “Didemnitutus” denotes a bacterium protected by a member of the family *Didemnidae*, and “mandela” is an allusion to the collection site, near the Nelson Mandela Bay municipality in South Africa. The closest relative to the symbiont that has a publicly available genome sequence is *Opitutus* sp. strain GAS368 (92% 16S rRNA sequence identity). According to the sequence cutoffs proposed by Yarza et al. (19), this level of identity would be consistent with a new genus in the family *Opitutaceae*.

10.1128/mSystems.00096-17.1 FIG S1  Approximately maximum-likelihood tree generated by FastTreeMP from 31 concatenated single-copy marker gene protein sequences from the “*Ca*. Didemnitutus mandela” genome and 1,068 other reference genomes (species outside the PVC superphylum and *Elusimicrobia* not shown). Bootstrap proportions greater than 70% are expressed to the left of each node as a percentage of 1,000 replicates. Download FIG S1, EPS file, 0.9 MB. Copyright © 2017 Lopera et al. 2017 Lopera et al. This content is distributed under the terms of the Creative Commons Attribution 4.0 International license.

Given that the symbiont “*Ca*. Didemnitutus mandela” 16S rRNA sequence could not be amplified with standard MiSeq universal primers, we were not able to directly quantify the abundance of “*Ca*. Didemnitutus mandela” relative to other bacteria represented in the metagenome of the tunicate consortium. We obtained a relatively low number of 16S reads from the zooid amplification product, compared to that of the tunic, potentially due to a low copy number of amplifiable 16S genes in the zooids (Table 1). Degenerate ketosynthase (KS) primers targeted to polyketide synthase (PKS) genes were then used to quantify the levels of the *mnd* BGC and other pathways. This revealed that 96.8% of reads in the zooid fraction originated from *mnd*, as well as 50.5% of reads from the tunic. A very low number of the non-*mnd* reads were identified as being from *trans*-AT-type PKS pathways (Fig. 1E), and therefore, *mnd* is the only detectable pathway capable of making the mandelalides (see also details of the proposed biosynthetic scheme, below).

### Multiple copies of the *mnd* biosynthetic gene cluster are maintained in the symbiont genome despite streamlining.

**FIG 2:**
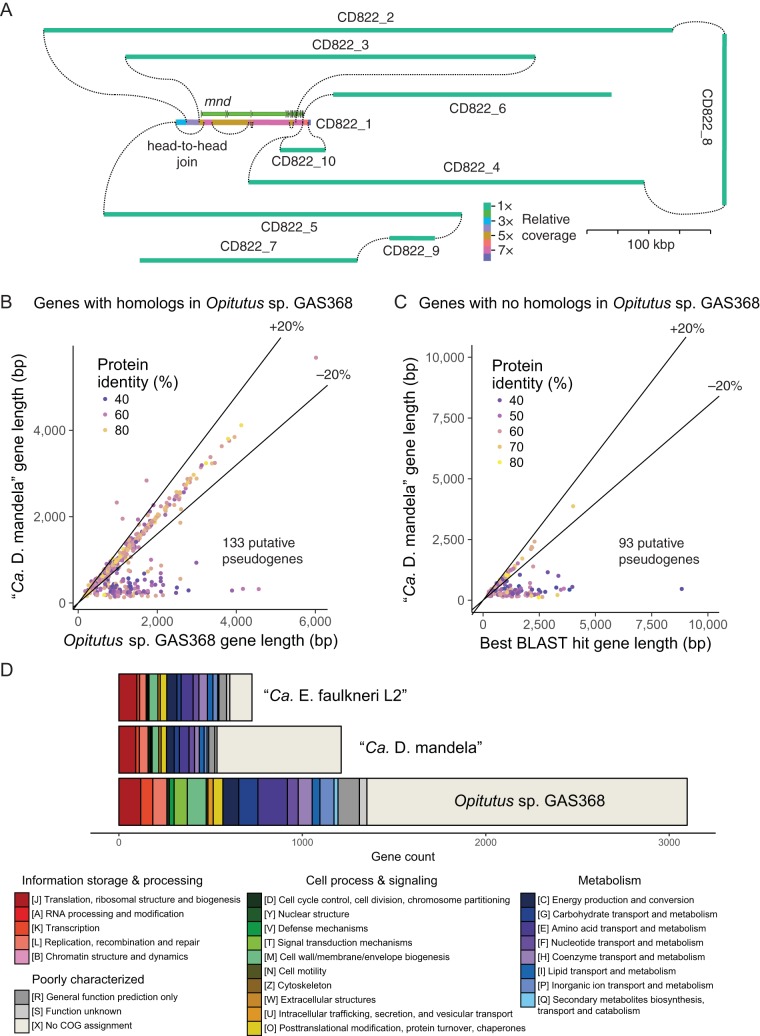
(A) Scale map of connections between contigs in Ver_v2 suggested by the alignment of paired-end Illumina reads (insert size, ~300 bp). Colors denote different relative coverages. Deletions are suggested by joins between distal regions in the *mnd* gene cluster. While deleted regions have 5× coverage, the rest of *mnd* has 7× coverage, indicating that deletions occur in 2/7 repeats. (B) Comparison of gene length in “*Ca*. Didemnitutus mandela” and *Opitutus* sp. GAS368. Genes <80% of the length of the *Opitutus* sp. GAS368 homolog are putative pseudogenes. (C) For genes without a homolog in *Opitutus* sp. GAS368, length is compared to their closest BLASTP hit. (D) Analysis of COG gene categories in “*Ca*. Endolissoclinum faulkneri,” “*Ca*. Didemnitutus mandela,” and *Opitutus* sp. GAS368, for genes that are not putative pseudogenes.

In order to resolve the repeat structure within Ver_v2, we examined the read coverage of all contigs in the assembly. We aligned paired-end reads from both the zooid and tunic metagenomes to Ver_v2 contigs, to detect joins suggested by read pairs that aligned to different contigs. Joins were considered between the ends of contigs and also to middle regions, especially where abrupt changes in read coverage suggested that the assembler had joined two repeats of differing copy number (Fig. 2A). The high-coverage region of the genome includes the *mnd* pathway except for *mndR*, which has a relative coverage of 1× and resides on contig CD822_6. Sections of the repeat region range in relative coverage from 3× to 8× because the ends of these regions are variable, with several relatively rare deletions within the *mnd* cluster. The deleted regions have 5× coverage, and the rest of the *mnd* pathway has 7× coverage, suggesting either that there are two *mnd* repeats with three deletions each or that these deletions are evenly distributed among each of the seven repeats, each occurring twice. There is evidence of multiple alternate connections between both ends of the repeat region and 1× contigs, supporting the notion that the *mnd* pathway is repeated multiple times in the genome and not simply embedded in a different genome. A number of connections could also be made between 1× contigs, suggesting five 1× scaffolds, several of which are joined to *mnd* at both ends (Fig. 2A). With only two loose ends in the connection map, it appears that the majority of the genome is represented in the assembly. Our estimation of 94.2% completeness from single-copy marker genes, therefore, might be due to a bona fide absence of a small number of markers, potentially due to genome reduction. We have observed much lower apparent completeness values in the complete chromosome of a more extensively eroded genome (15). If the genome consists of a single circular chromosome, our findings mean that there are five repeat regions in the genome, with some adjacent *mnd* repeats. Consistent with this notion, paired-end alignments showed evidence of head-to-head connections at least two *mnd* repeats.

Factoring in the copy number of repeat regions, the intact chromosome of “*Ca*. Didemnitutus mandela” was calculated to be 2.68 Mbp in size. The coding density of the repeat region is 84.9%, with an average gene size of 1,574 bp, whereas the coding density of the 1× contigs is 64.3%, with an average gene size of 470 bp. The copy number of each *mnd* gene was calculated from the total number of repeats in which the respective gene is not truncated, and such intact genes should occupy 514,374 bp, accounting for 19.2% of the genome and 25.8% of its entire coding capacity. We analyzed a set of symbiont genomes and genomes of free-living bacteria (Table S1) and found that several facultative and transitional symbionts have a similar or higher fraction of repeats. However, in all other cases, there seemed to be a general proliferation of many repeat loci, with few long repeats, and “*Ca*. Didemnitutus mandela” is unique in having repetition of genes from an entire pathway. The lower coding density within the 1× regions of the “*Ca*. Didemnitutus mandela” genome suggested sequence degradation characteristic of symbionts shortly after a change in lifestyle such as restriction to a particular host or a switch to an intracellular habitat (4). Therefore, we examined the gene inventory beyond *mnd*.

10.1128/mSystems.00096-17.9 TABLE S1  Comparison of repeat structure in symbionts and free-living bacteria. Download TABLE S1, PDF file, 0.1 MB. Copyright © 2017 Lopera et al. 2017 Lopera et al. This content is distributed under the terms of the Creative Commons Attribution 4.0 International license.

Out of 2,864 predicted protein-coding genes in “*Ca*. Didemnitutus mandela,” only 780 were found to have homologs in *Opitutus* sp. GAS368. A further 162 genes had BLAST hits in the NCBI NR database, and the remainder (*n* = 1,922) were found to have much shorter average length and slightly lower GC content (Table 3). Of the “*Ca*. Didemnitutus mandela” genes found to be homologous to genes in *Opitutus* sp. GAS368, 133 were significantly truncated (>20%), suggesting that they are pseudogenes (20) (Fig. 2B). Of the remaining genes with BLAST hits, 93 of them were truncated more than 20% compared to their best BLAST hit and are counted here as putative pseudogenes (Fig. 2C). The genes that are truncated may reflect functions that are under reduced selection for retention in the symbiotic relationship (Table S2); for example, there are many putative pseudogenes involved in lipopolysaccharide (LPS) biosynthesis. Key enzymes involved in the biosynthesis of the amino acids isoleucine, valine, leucine, proline, and tryptophan are also truncated. All but one of these amino acids cannot be synthesized by eukaryotic organisms (21). Consequently, it is unlikely that “*Ca*. Didemnitutus mandela” serves a nutritional function for the host organism. Putative pseudogenes were also found in the pathways for some cofactors, including riboflavin and folate.

10.1128/mSystems.00096-17.10 TABLE S2  Putative pseudogenes with functional annotations in the “*Ca*. Didemnitutus mandela” genome. Download TABLE S2, PDF file, 0.1 MB. Copyright © 2017 Lopera et al. 2017 Lopera et al. This content is distributed under the terms of the Creative Commons Attribution 4.0 International license.

**TABLE 3:** Characteristics of genes and intergenic sequences in the “*Ca*. Didemnitutus mandela” genome

Type of sequence in “*Ca*. Didemnitutus mandela”	Avg length (bp)	GC%	*n*
Gene with homolog in *Opitutus* sp. GAS368	981	53.9	780
Gene with no homolog in *Opitutus* sp. GAS368 but BLAST hit in NR	993	55.0	162
Gene with no homolog in *Opitutus* sp. GAS368, no BLAST hit in NR	259	51.1	1,922
Intergenic sequence	295	49.0	2,794

The number of genes with annotated functions in “*Ca*. Didemnitutus mandela” is on a par with “*Ca*. Endolissoclinum faulkneri,” an intracellular symbiont of *L. patella* and source of the patellazoles, cytotoxic polyketides (5, 6) (Fig. 2D). However, a number of factors suggest that “*Ca*. Endolissoclinum faulkneri” is in a more advanced state of genome reduction than “*Ca*. Didemnitutus mandela.” “*Ca*. Endolissoclinum faulkneri” has a lower coding density than “*Ca*. Didemnitutus mandela” (Table 4), and the intergenic regions of the former are degraded to the point that there are few recognizable pseudogenes and there is a pronounced AT-skew compared to coding regions (5, 6) not seen in “*Ca*. Didemnitutus mandela.” “*Ca*. Didemnitutus mandela” also possesses some key genes that “*Ca*. Endolissoclinum faulkneri” has lost, including *dnaA* and *ftsZ*, which are central to chromosome replication and cellular division, respectively. This suggests that “*Ca*. Didemnitutus mandela” maintains more control over these processes than “*Ca*. Endolissoclinum faulkneri.”

**TABLE 4:** Comparison of “*Ca*. Didemnitutus mandela,” *Opitutus* sp. GAS368, and “*Ca*. Endolissoclinum faulkneri” L2 genomes

Characteristic	Value for genome:		
	“*Ca*. Didemnitutus mandela”	*Opitutus* sp. GAS368	“*Ca*. Endolissoclinum faulkneri” L2
Genome length, Mbp	2.68	4.15	1.48
% coding (length)	69.0^a^	89.4	57.2
GC%	51.9	65.7	34.1^b^
No. of nonhypothetical genes	654	2,016	688
No. of hypothetical genes	2,031	1,389	95
No. of pseudogenes	226	183	5
% secondary metabolism (fraction of coding length)	25.8	3.2	10.2

a Repeat region density, 84.9%; 1× contigs, 63.4%.

b Coding regions are 40.9% GC, whereas noncoding regions are 24.7% GC.

We did, however, find that several DNA repair pathways have deficiencies in the “*Ca*. Didemnitutus mandela” genome. The nucleotide excision repair pathway is complete, as is the mismatch repair pathway (in contrast to “*Ca*. Endolissoclinum faulkneri”). However, the base excision repair pathway is missing two DNA glycosylases (*alkA* and *tag*) responsible for removing 3-methyladenine adducts, one of which is still present in “*Ca*. Endolissoclinum faulkneri.” Additionally, the homologous recombination system is missing several key genes (*recB*, *recF*, *recN*, and *recQ*). The loss of these genes should preclude both RecBCD-dependent and RecBCD-independent homologous recombination, as well as incorporation of horizontally transferred DNA into the chromosome (22). Taken together, this pattern of maintenance and loss suggests that “*Ca*. Didemnitutus mandela” is still able to copy its genome with fidelity but is likely vulnerable to strand breaks due to impaired homologous recombination systems. No transposases, integrases, or restriction-modification or phage genes (3) were annotated, and “*Ca*. Didemnitutus mandela” does not have a nonhomologous end-joining system. Therefore, it would appear that further genome rearrangement is possible only through RecA-independent “illegitimate recombination” (3) during chromosome replication.

Secondary metabolism is by definition more variable among close relatives than are central functions (23), and it is thought that BGCs are often disseminated via HGTs (24). Since HGTs are likely no longer possible in “*Ca*. Didemnitutus mandela,” and genome rearrangements are probably rare at this stage, the *mnd* cluster was probably obtained before homologous recombination was lost, with duplication occurring shortly after acquisition. Notably, the cluster repeats have been present long enough to allow divergence through deletions and the accumulation of a small number of single nucleotide polymorphisms (SNPs) (Fig. S2). Consistent with this timeline, we found that the codon adaptation index (CAI) (25) of *mnd* genes is not significantly different from those of other genes with annotated function (Fig. S3). Interestingly, we found that the CAIs of both pseudogenes and hypothetical genes are significantly different from those of genes with annotated functions, suggesting that these ORFs are degraded with concomitantly reduced codon selection.

10.1128/mSystems.00096-17.2 FIG S2  SNPs identified within the *mnd* pathway (*mnd* genes are green, other protein coding genes are gray, and tRNAs are magenta). Read coverage is plotted above the contig. Download FIG S2, EPS file, 1.1 MB. Copyright © 2017 Lopera et al. 2017 Lopera et al. This content is distributed under the terms of the Creative Commons Attribution 4.0 International license.

10.1128/mSystems.00096-17.3 FIG S3  (A) Box plots of codon adaptation index (CAI) of different groups of genes within the “*Ca*. Didemnitutus mandela” genome. (B) Individual *P* values for pairwise comparisons of the groups compared through one-way ANOVA and Tukey HSD. Significant *P* values (*P* < 0.05) are highlighted in red. Annotated genes are not significantly different in codon usage from *mnd* genes. However, both pseudogenes and hypothetical genes are significantly different from annotated genes in this respect. Download FIG S3, EPS file, 0.9 MB. Copyright © 2017 Lopera et al. 2017 Lopera et al. This content is distributed under the terms of the Creative Commons Attribution 4.0 International license.

### Model for mandelalide biosynthesis by *mnd*.

**FIG 3:**
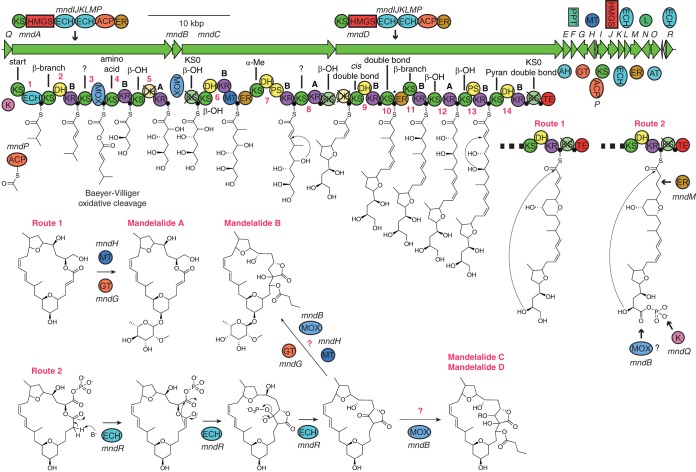
Proposed mandelalide biosynthetic pathway. The *trans*-AT PKS pathway consists of 14 modules with proposed chain shortening mediated by a monooxygenase. Butyrolactone formation (as in mandelalides B to K) is proposed through the action of MndR, a homolog of Dieckmann cyclase MenB. Modules are numbered in red, and predicted substrates (Fig. S4) are shown next to the respective KS domain. A cross indicates domains predicted to be catalytically inactive. Abbreviations: ACP, acyl carrier protein, also denoted by a filled black circle; AH, acylhydrolase; AT, acyltransferase; DH, dehydratase; ECH, enoyl-CoA reductase; ER, enoylreductase; GT, glycosyltransferase; HMGS, 3-hydroxy-3-methylglutaryl-CoA synthase; K, kinase; KR, ketoreductase; KS, ketosynthase; L, phospholipase; MOX, flavin monooxygenase; MT, *C*-methyltransferase; PPT, phosphopantetheinyltransferase; PS, pyran synthase; TE, thioesterase.

Our model for the biosynthesis of mandelalides is shown in Fig. 3. The pathway consists of three large PKS proteins and 15 accessory proteins. These accessory proteins include a phosphopantetheinyltransferase (PPT, MndF), required to postranslationally modify acyl carrier protein (ACP) domains within the PKS with a phosphopantetheine arm (26, 27), and a *trans*-acting acyltransferase (AT, MndO), which is responsible for loading malonyl-*S*-coenzyme A (CoA) extender units onto these phosphopantetheine arms (13, 14). The *mnd* cluster also contains a suite of proteins predicted to install the β-methyl at carbon 11 (MndIJKLMP) (28); a glycosyltransferase (GT, MndG), which may attach a sugar unit to the polyketide core structure; and a methyltransferase (MT, MndH), which could supply the *O*-methyl group to the sugar units observed in the known mandelalide structures (8, 9).

10.1128/mSystems.00096-17.4 FIG S4  Expansions of clades containing *mnd* KS domains in an approximately maximum-likelihood tree made from 665 KS domain sequences. Colors in each clade correspond to the chemical structures shown immediately to its right side. Download FIG S4, PDF file, 1.6 MB. Copyright © 2017 Lopera et al. 2017 Lopera et al. This content is distributed under the terms of the Creative Commons Attribution 4.0 International license.

Type I PKS proteins, such as MndACD, cause the synthesis of specific structures through the presence or absence of specific enzymatic domains within “modules,” which each add a C_2_ unit, analogous to an assembly line (29). This process results from repeated Claisen condensation of a ketosynthase (KS)-bound thioester intermediate onto ACP-bound malonate to make a β-keto thioester with the loss of CO_2_. If the module additionally contains a ketoreductase (KR) domain, then the β-position is reduced to a hydroxyl moiety. Inclusion of a dehydratase (DH) domain in addition to a KR results in an α-β double bond, and the presence of an enoylreductase (ER) domain with DH and KR results in complete reduction of the β-position to give an alkyl chain. Many other exotic variants of module structure are known in *trans*-AT systems (13, 14), and KS domains in these pathways have diversified to accept specific substrate structures (30–32). Through phylogenetic analysis, substrate specificity was predicted for the majority of *mnd* KS domains (Fig. S4) and found to be almost completely congruent with our proposed biosynthetic scheme (Fig. 3). In this case, the order of PKS proteins appears to be colinear with the gene order, as the first KS of MndA is closely related to others in starter modules of *trans*-AT PKS pathways and because MndD contains a thioesterase (TE) domain for cleaving and macrocyclization of the final PKS product.

Similar to other *trans*-AT pathways, several domains in MndACD were predicted to be nonfunctional due to disrupted catalytic residues or truncated sequences (Fig. S5). However, the three PKS proteins still contain 14 extending modules—three more than would be needed to make the mandelalides (Fig. 1B). We rationalize this discrepancy by proposing that the monooxygenase (MOX) domain in MndA carries out a Baeyer-Villiger-type oxidation, thereby effecting oxidative cleavage of the intermediate. The following KS is related to amino-acid-accepting KSs, even though it does not follow a nonribosomal peptide synthetase (NRPS) module. The predicted intermediate accepted by this KS is a hydroxy acid (glycolic acid), similar to the amino acid glycine except that the glycine nitrogen is replaced by an oxygen. A similar mechanism of chain cleavage is thought to occur in the pederin (33) and diaphorin (34) pathways. This mechanism would also generate the apparent starter unit for mandelalide A, 3,4-dihydroxybutanoic acid. There are a number of other features that are consistent with the final mandelalide structures. In particular, there are two modules containing pyran synthase (PS) domains (35) (modules 7 and 12, Fig. S6), which are the correct distance apart to install both the tetrahydrofuran (THF) and tetrahydropyran (THP) rings in mandelalide A. Module 10 also contains a specialized β-branching ACP (36), which would cause the installation of a β-methyl in the expected location next to the THP (Fig. S5). Additionally, based on previous findings (37), KR domains were analyzed to predict the configuration of installed hydroxyl groups (Fig. S5). In all cases, the predicted configurations match configurations confirmed in mandelalide A by total synthesis (10, 38–42).

10.1128/mSystems.00096-17.5 FIG S5  (A) Alignment of *mnd* KS domains, with the CHH catalytic triad highlighted. The domains where this triad is disrupted are predicted to be catalytically inactive. (B) Alignment of *mnd* KR domains, along with two from the erythromycin biosynthetic gene cluster to allow comparison to previous alignments. The NADPH-binding site (GxxxGxG) is highlighted, as is the LDD motif that is thought to control product stereoconfiguration. The catalytic triad (KSY) is also highlighted. (C) Alignment of *mnd* DH domains. Highlighted are the conserved motifs HxxxGxxxxP and DxxxQ, which are involved in catalysis. (D) Alignment of *mnd* ACP domains, with the extender unit attachment point serine highlighted. In addition, residues characteristic of β-branching ACPs in MndD_ACP3 are shown in red. Download FIG S5, PDF file, 0.3 MB. Copyright © 2017 Lopera et al. 2017 Lopera et al. This content is distributed under the terms of the Creative Commons Attribution 4.0 International license.

10.1128/mSystems.00096-17.6 FIG S6  Approximately maximum-likelihood tree based on DH and PS domains. The PS domains form a distinct clade, as shown previously (35). Download FIG S6, EPS file, 1.9 MB. Copyright © 2017 Lopera et al. 2017 Lopera et al. This content is distributed under the terms of the Creative Commons Attribution 4.0 International license.

Often, *trans*-AT PKS proteins deviate from strict gene and domain colinearity (13, 14); for example, in the *mnd* pathway modules 8 and 13 lack DH domains even though they should introduce double bonds. These modules may utilize DH domains in the following module, similar to many other *trans*-AT pathways, such as those that produce bacillaene, calyculin, and oxazolamycin (13, 14). Module 13 contains a PS domain, which is related to dehydratases but lacks key catalytic residues (35). This pattern is consistent with the structures of the mandelalides, but the reaction requires the installation of an α-β double bond, which could be installed by the DH in module 14. We also found that the KR domain in module 5 has a noncanonical catalytic triad—KSH instead of KSY (43) (Fig. S5). This mutation appears to be rare; the only other example that could be found is the KR domain of the ena5920 protein within the pathway for enacycloxin (44). This KR domain is annotated as functional, and we propose that histidine likely fulfills the same proton source role as tyrosine during generation of the β-keto group of the substrate (Fig. S7).

10.1128/mSystems.00096-17.7 FIG S7  (A) Canonical mechanism of KR domains with catalytic triad KSY. (B) Proposed mechanism of functional KR domains with catalytic triad KSH. Download FIG S7, EPS file, 2.2 MB. Copyright © 2017 Lopera et al. 2017 Lopera et al. This content is distributed under the terms of the Creative Commons Attribution 4.0 International license.

A compelling mystery in the biosynthesis of mandelalides is the mechanism by which a butyrolactone is installed in mandelalides B to K at the point of macrocyclization, which would require both ester and C–C bond formation. A further question is how the pathway produces butyrolactone-containing mandelalides alongside mandelalide A, which lacks this moiety. We propose that the butyrolactone is generated by MndR, a homolog of crotonase superfamily member MenB. For both mandelalide A-type and B-type compounds, we predict that the thioesterase of MndD produces an initial macrocycle. MenB catalyzes a Dieckmann cyclization to produce dihydroxynaphthoyl-CoA in the vitamin K biosynthesis pathway (45, 46), and we predict that MndR could analogously form the lactone C–C bond in a Dieckmann reaction (Fig. 3). In order to allow for the action of MndR, we propose that the terminal hydroxyl is oxidized to a carboxylic acid, which is then phosphorylated by kinase MndQ, so as to activate the carbonyl to nucleophilic attack. Additionally, the α-β double bond could be removed by *trans*-acting ER MndM, to allow the formation of an enolate which can attack the phosphoester.

### The mandelalide-containing *Lissoclinum* sp. is a novel species of tunicate.

**FIG 4:**
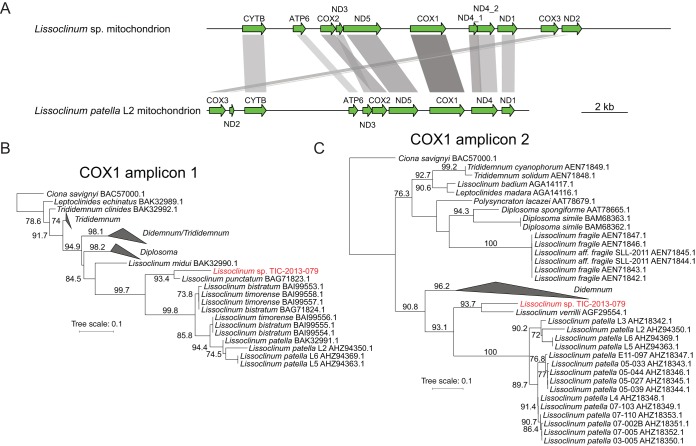
(A) Scale map of *Lissoclinum* sp. mitochondrion genome and comparison to the genome of the *Lissoclinum patella* L2 mitochondrion. (B and C) Approximately maximum-likelihood trees based on two nonoverlapping amplicons in the mitochondrial cytochrome *c* oxidase I (COX1) gene. Bootstrap proportions greater than 70% are expressed to the left of each node as a percentage of 1,000 replicates. Sequence identities of the *Lissoclinum* sp. COX1 gene to its close relatives are shown in Fig. S8.

A BLAST search using *L. patella* cytochrome *c* oxidase 1 (COX1) protein sequences as queries identified a contig in the metagenomics assembly that appeared to represent the majority of the host mitochondrial genome (Fig. 4). This contig is 20.7 kbp in length and contains all the protein-coding genes previously identified in the *L. patella* mitochondrial genome (47). Comparison of the coding sequences in *Lissoclinum* sp. and *L. patella* L2 (Fig. 4A) revealed several genes that appeared to be shorter in L2. The NADH dehydrogenase subunit 4 (ND4) gene appears to be disrupted by a frameshift in *Lissoclinum* sp., but this disruption could be an artifact of sequencing errors due to low sequence complexity and prevalent homopolymers within the contig. Assuming a circular chromosome, the gene order in *Lissoclinum* sp. is very similar to the gene order in the *L. patella* L2 mitochondrial genome, except for a swap in the positions of ATP synthase F_o_ subunit 6 (ATP6) and cytochrome *c* oxidase subunit 2 (COX2). Both the mitochondrial genomes of *Lissoclinum* sp. and *L. patella* L2 are very low in GC content (12.4% and 21.2%, respectively). The resulting low complexity of the sequences makes it difficult to detect rRNA genes. Previously, we suggested that the rRNA genes are in the space between the CYTB and ATP6 genes in *L. patella* L2 (47). A corresponding space without detectable CDSs is present in the *Lissoclinum* sp. mitochondrial genome, after the ND2 gene, potentially signifying a second rearrangement (Fig. 4A). It has been suggested previously that gene order in tunicate mitochondrial genomes could be used as a phylogenetic signal (48). The gene rearrangements observed therefore suggest that *Lissoclinum* sp. is phylogenetically distinct from *Lissoclinum patella*.

Sections of the COX1 gene have been used for molecular barcoding in tunicates, but unfortunately, two nonoverlapping regions of the gene have been employed (47). In two phylogenetic trees based on COX1 protein sequence (Fig. 4B and C), the mandelalide-containing *Lissoclinum* sp. appears to be a divergent *Lissoclinum*, distinct from a major group that includes *L. patella*, *Lissoclinum bistratum*, and *Lissoclinum timorense*. The closest relatives to *Lissoclinum* sp. are *Lissoclinum punctatum* and *Lissoclinum verrilli*. The *Lissoclinum* sp. COX1 protein sequence has 72 to 75% identity to *L. patella* specimens, 76 to 78% identity to *L. bistratum* and *L. timorense*, and 82% identity to its closest relative, *L. punctatum* (Fig. S8). The divergence of *Lissoclinum* sp. and *L. punctatum* is on par with the evolutionary distance between different subpopulations of *L. patella* that we believe represent multiple cryptic species with a common ancestor that existed 6 to 31 million years ago (6). Therefore, the mandelalide-containing *Lissoclinum* sp. may be a novel species of tunicate in the family *Didemnidae*.

10.1128/mSystems.00096-17.8 FIG S8  Matrices showing pairwise amino acid identities for the full-length *Lissoclinum* sp. COX1 gene and other COX1 sequences from the NCBI database. Download FIG S8, PDF file, 1.1 MB. Copyright © 2017 Lopera et al. 2017 Lopera et al. This content is distributed under the terms of the Creative Commons Attribution 4.0 International license.

## DISCUSSION

Culture-independent sequencing has revealed evidence of the phylum *Verrucomicrobia* in a variety of terrestrial and marine environments, although relatively few species have been isolated and/or sequenced (49). However, both free-living and symbiotic verrucomicrobial species are known. For instance, *Akkermansia muciniphila* is a prevalent member of the human gut microbiota that degrades mucins (50). Intracellular and genome-reduced *Verrucomicrobia* members are also known, such as “*Candidatus* Xiphinematobacter” in nematodes (51) and “*Candidatus* Nucleococcus,” which lives inside the nucleus of protists in the termite gut (52). In contrast to “*Ca*. Didemnitutus mandela,” these and related symbionts have yet to be associated with what appears to be highly defensive functions by virtue of toxin biosynthesis. Secondary metabolite pathways have been noted in the genomes of *Verrucomicrobia* (53, 54), but to the best of our knowledge “*Ca*. Didemnitutus mandela” is the only species in this phylum that has been linked to secondary metabolites that have been isolated, structurally characterized, and shown to be potently cytotoxic. Our findings here reiterate the sentiment that uncultured bacterial lineages may be a prolific source of bioactive natural products for drug discovery.

The mandelalides were previously found to be potent cytotoxins (8, 9) and may therefore serve as chemical defenses for the tunicate host, similar to what has been noted for the patellazoles in *L. patella* (5). The structures of mandelalides B to K are unique among cyclic polyketides, which invariably are cyclized through formation of an ester bond (29). The macrocycles of mandelalides B to K are formed through both an ester and C–C bonds that constitute a butyrolactone not found in mandelalides A and L. To the best of our knowledge, the only comparable macrocyclization occurs in the biosynthesis of lankacidin, in which an amine oxidase produces an imine for attack by an acidic carbon between two carbonyl groups, thus forming a C–C bond (55). In both cases, the resulting C–C macrocycle may be more pharmacokinetically stable by virtue of its resistance to circulating esterases *in vivo*, which break down ester-containing drugs and thus limit efficacy and duration of action (56). Accordingly, further study of the biochemical mechanisms of these macrocyclizations is likely to aid the design of pharmacokinetically stable polyketide drugs. In the case of the *mnd* pathway, confirmation of the mechanism of butyrolactone production would require heterologous expression of *mnd* genes and characterization of their biochemical activities *in vitro*.

The duplication of a very long gene cluster such as *mnd* in “*Ca*. Didemnitutus mandela” has not been observed in nature, to the best of our knowledge, especially in a genome-reduced symbiont. As gene amplification is a rapid and common process (57), it is reasonable to suppose that such duplications of secondary metabolite pathways do occur in nature given the right selective environment. Indeed, the duplication or amplification of pathways has been observed in industrial actinomycete strains that have been heavily mutagenized and selected for higher-level production (58, 59). A similar effect has been achieved in actinomycetes through purposeful pathway amplification (60, 61). This suggests that *mnd* is under strong selection, in a manner similar to the *trpEG* genes in the aphid endosymbiont *Buchnera aphidicola*, which provide tryptophan to the host (62). *B. aphidicola* has been an endosymbiont for ~150 million years (63) and now has a very small genome (~640 kbp). Remarkably, despite extreme genome reduction, some strains harbor a plasmid with multiple copies of *trpEG*, although there are often pseudogenes among the copies (64). In the early stages of the symbiosis, the plasmid location of these genes may have increased gene dosage and tryptophan production. However, at some later point, the *Buchnera* chromosome became polyploid, and there is evidence of back-transfer of these genes to the chromosome in some lineages (65). These back-transfers and *trpEG* copy number variants are the only recombination events known to have occurred since the divergence of extant *Buchnera* strains, which lack RecA but maintain RecBCD (65).

The genome of “*Ca*. Didemnitutus mandela” shows signs of degradation and reduction consistent with host restriction, although this change in lifestyle is likely to have happened relatively recently. With only one sequenced strain of “*Ca*. Didemnitutus mandela” and the paucity of known close relatives to either the symbiont or host, it is difficult to date the symbiosis, except through loose comparisons to unrelated symbiotic systems. Mandelalide-producing tunicates have been found in only one place on Earth, and so, more complete investigation of the evolution of “*Ca*. Didemnitutus mandela” is currently very challenging. The erosion of the “*Ca*. Didemnitutus mandela” genome is not as severe as in *B. aphidicola* (~150 million years) (63) or “*Ca*. Endolissoclinum faulkneri,” which has been an intracellular symbiont for at least ~6 to 31 million years (6). However, the “*Ca*. Didemnitutus mandela” genome contains fewer recognizable pseudogenes relative to a very recent symbiont such as *Sodalis glossinidius*, which diverged from a close free-living relative ~30,000 years ago (66) and has 972 pseudogenes in its genome (67). The current repeat structure of *mnd* in the chromosome is also likely not recent, as we predict “*Ca*. Didemnitutus mandela” to be recombination deficient, and at this point, the gene order is likely fixed. Accordingly, we found the codon usage in *mnd* to be consistent with the rest of the genome. We found that only a small number of mutations had accumulated in *mnd* since duplication, perhaps because DNA repair pathways remain largely intact. Nevertheless, with the cessation of recombination and the segregation of small populations within individual hosts, degradation continues through a process known as “Muller’s ratchet” (68). Mutations and deletions that are not outright lethal tend to become irreversible through population bottlenecks and the inaccessibility of HGT or recombination events. Due to the deletion and AT mutation bias of bacteria, along with weakened selection caused by small effective populations (1), nonfunctional pseudogenes and intergenic sequences will be lost quickly and the sequence of essential genes will drift. This process will accelerate when DNA repair pathways become compromised. The copies of *mnd* are likely to continue diverging from each other as their sequences degrade, before individual gene copies become nonfunctional pseudogenes and are deleted. The result of such a process would appear similar to the genome of “*Ca*. Endolissoclinum faulkneri,” where genes from a single pathway are fragmented across many loci in the chromosome. In nonsymbiotic bacteria, there is a strong tendency for the genes of secondary metabolite pathways to remain clustered on a contiguous region of a chromosome or plasmid (7), and it is thought that this colocalization is advantageous in coregulating genes and operons (69). It has been suggested that clustering aids HGTs in the “selfish operon” hypothesis (70). However, such events may be quite rare (24) and therefore have little influence on selective pressures to maintain clustering. We have observed that secondary metabolite BGCs in symbionts tend to be fragmented more often than expected (7). A potential explanation for this fragmentation is a reduced need for fine regulation of a product that is always needed (e.g., for defense), in an environment where production has little survival cost since nutritional needs are met by the host. Our results here suggest that biosynthetic pathway fragmentation in symbionts could also arise through strong selection for high production at the onset of symbiosis, causing pathway duplication prior to genome degradation.

## MATERIALS AND METHODS

### Tunicate collection, preservation, and DNA extraction.

A specimen of *Lissoclinum* sp. was collected at 33°59′55″S, 25°42′43″E on 7 July 2013 from White Sands Reef in Algoa Bay, Eastern Cape Province, South Africa, by scuba at an approximate depth of 18 m. A voucher specimen is maintained with the designation TIC-2013-079 at the South African Institute for Aquatic Biology (SAIAB), Grahamstown, South Africa. Part of the animal was preserved in RNAlater at −80°C. The remainder was used for natural product isolation studies reported elsewhere (9). The preserved tissue was later dissected to separate zooids from the tunic, and DNA was extracted as previously described (5).

### Illumina sequencing and metagenome assembly.

Illumina TruSeq libraries were prepared with ~300-bp inserts from DNA obtained from zooids and tunic of *Lissoclinum* sp. Libraries were sequenced using an Illumina HiSeq 2000 sequencer in multiple 101-bp paired-end runs. Sequence yields are shown in Table 1. Contaminating adaptor sequences were removed with Trimmomatic (71), and the trimmed reads were assembled with metaSPAdes (72).

### Construction of the draft “*Ca*. Didemnitutus mandela” genome.

Contigs in the metagenomic assembly were classified taxonomically from their predicted ORFs as previously described (15, 16). All contigs classified as belonging to the phylum *Verrucomicrobia* were separated. Trimmomatic-filtered reads from both zooid and tunic fractions were aligned to these contigs with Bowtie 2 (73) (using the --very-sensitive option), and the aligned reads were assembled separately with SPAdes (74), using the --careful parameter. To identify potential connections between contigs and repeats, reads were realigned to contigs or derived sequences using Bowtie 2 (73) and the cytoscapeviz.pl script, part of the Multimetagenome package (75), was run on the alignment. Connections were visualized in Cytoscape (76). The Ver_v2 assembly was annotated with Prokka (77).

### Single-copy marker gene analysis.

A set of 139 single-copy marker genes was identified using HMM profiles and cutoffs determined by Rinke et al. (17). The number of different marker genes, expressed as a percentage of 139, was used to estimate bacterial genome completeness. The number of different marker genes unique in a bin, expressed as a percentage of 139, was used to estimate genome purity.

### Amplicon sequencing.

An ~430-bp section of 16S rRNA genes was amplified from DNA extracts using primers S-D-Bact-0341-b-S-17 and S-D-Bact-0785-a-A-21 (78), and an ~700-bp section of ketosynthase domains was amplified using primers KS-F and KS-R (79). In both cases, additional custom 5′ ends were added to primers, specific to each sample, including MiSeq adaptor sequences and a sample-identifying barcode sequence. Pooled amplicons were sequenced on an Illumina MiSeq instrument in a 251-bp paired-end run. For each sample, 16S and KS amplicons were dereplicated by identifying the respective primer sequences in the reads. The forward KS reads were used as queries in BLASTN searches against the *mnd* pathway, and reads with ≥97% identity and alignment of >90% of the read length were counted as *mnd* reads. To determine which KS reads were likely part of *trans*-AT PKS pathways, the forward reads were used as queries in a BLASTX search against the NCBI NR database, using the accelerated BLAST implementation DIAMOND (80). A list of proteins from *trans*-AT PKS pathways containing KS domains was compiled, and the accession numbers from this list were used to identify KS reads where one or more of the first 500 BLASTX hits were in the *trans*-AT list. These reads were counted as *trans*-AT KS reads.

### Construction of phylogenetic trees.

Sequences used to make phylogenetic trees were aligned with either ClustalX (81) (small data sets) or Clustal Omega (82) (large data sets), except for the 16S rRNA tree, which used aligned sequences downloaded from the Ribosomal Database Project server (83). Alignments were inspected manually and trimmed before trees were constructed with FastTreeMP (84). The parameters “-slow -spr 5 -mlacc 3 -gamma -gtr -nt” and “-slow -spr 10 -mlacc 3 -bionj -gamma” were used to produce the nucleotide and protein trees, respectively. Trees were visualized on the Interactive Tree of Life server (85). To make the concatenated protein marker tree (see Fig. S1 in the supplemental material), marker protein sequences from the “*Ca*. Didemnitutus mandela” and *Opitutus* sp. GAS368 genomes were extracted with AMPHORA 2 (86). AMPHORA 2 was then used to make protein alignments with its reference database, and the corresponding tree was generated by concatenating the alignments, using only those genomes where all of the 31 markers shared by “*Ca*. Didemnitutus mandela” and *Opitutus* sp. GAS368 were present.

### Repeat analysis.

Representative genomes of bacteria with different lifestyles were assembled from examples listed in the work of Lo et al. (4), using only complete genomes, which should give a more accurate quantification of repeats versus draft genomes (Table S1). Additionally, *Opitutus* sp. GAS368 and “*Ca*. Endolissoclinum faulkneri” were included in the analysis. For each genome, repeat regions of >50 bp were identified by using Nucmer (87) to align the genome to itself. Duplicate and self-hits were removed before repeat regions were extracted for quantification of total length and number of loci, etc.

### Homolog analysis and identification of pseudogenes.

Predicted genes in the Ver_v2 assembly were used as queries in a BLASTP search against the NCBI NR database, using the accelerated BLAST implementation DIAMOND (80). The accession numbers of all annotated proteins in the *Opitutus* sp. GAS368 genome were obtained from NCBI, and the BLASTP table was searched for hits from this genome. If a protein from *Opitutus* sp. GAS368 was found in the first 100 hits, then the query protein was counted as having a homolog in this genome. R (88) was used to plot the comparison of homolog lengths (Fig. 2B). For proteins that did not have homologs in *Opitutus* sp. GAS368, the best BLASTP hit was instead used for comparison (Fig. 2C). To compare the *Opitutus* sp. GAS368 genome to that of “*Ca*. Didemnitutus mandela” (Table 4), the nucleotide sequence was reannotated in the same manner, with Prokka (77). To identify pseudogenes, predicted protein sequences were used as queries in a BLASTP search against the NCBI NR database, and then the annotated *Opitutus* sp. GAS368 proteins were removed from the results. Protein lengths were then compared to the respective best hit, and genes truncated by >20% compared to their best BLASTP hit were counted as putative pseudogenes. The secondary metabolite fraction of the *Opitutus* sp. GAS368 genome was calculated after searching for biosynthetic pathways with antiSMASH (89).

### Functional gene analysis.

The functional analysis shown in Fig. 2D was carried out as previously described (4, 90). Briefly, the protein sequences of nonpseudogenes from “*Ca*. Didemnitutus mandela,” “*Ca*. Endolissoclinum faulkneri,” and *Opitutus* sp. GAS368 were classified with the KEGG Automatic Annotation Server (KAAS) (91). The resulting KEGG classifications were converted to Cluster of Orthologous Group (COG) categories using mappings supplied through the KEGG database. Bar plots were created using R (88). The presence of specific functions was also assessed through analysis of BLASTP results in MEGAN (92) and with reference to the EcoCyc database (93).

### SNP detection.

Trimmed Illumina reads were aligned to the contig containing *mnd* (CD822_1), using Bowtie 2 (73) with the --very-sensitive parameter. The alignment was then loaded into Geneious (94) for SNP detection.

### CAI calculation.

Codon adaptation index (CAI) values were calculated according to the formula of Sharp and Li (25), using the *mnd* genes to calculate relative synonymous codon usage (RCSU) and *w* values (Fig. S3). The CAI values were separated into different gene categories and plotted as box plots in R, using the ggplot2 package (95). To test for statistically significant differences between groups, one-way analysis of variance (ANOVA) was carried out in R, using the aov function, followed by Tukey's honest significant difference (HSD) test for significance.

### Mitochondrion genome annotation and comparison to the *Lissoclinum patella* L2 mitochondrion.

An initial annotation was produced using Prokka (77) and manually inspected before additional genes were added, where appropriate, based on protein sequence similarity to other tunicate mitochondrial genes. Attempts were made to detect RNA genes with Rfam (96); however, none were found. Each gene was aligned to its counterpart in the *Lissoclinum patella* L2 mitochondrion genome using Clustal W (81), and the respective coordinates of the aligned regions were used to produce the diagram in Fig. 4A. The R library genoPlotR (http://genoplotr.r-forge.r-project.org) was used to plot both mitochondrial genomes to scale and the alignments.

### Accession number(s).

Raw Illumina reads were deposited in the Sequence Read Archive (SRA) under accession numbers SRR5712450 to SRR5712457. The draft assembly Ver_v2 of the “*Ca*. Didemnitutus mandela” genome was deposited in GenBank under accession number NJAL00000000. The mitochondrial genome of *Lissoclinum* sp. was deposited in GenBank under accession number MF573328.
