# Supplementary material for: Increased Biosynthetic Gene Dosage in a Genome-Reduced Defensive Bacterial Symbiont
Source: mSystems. 2017 Nov 21;2(6):e00096-17. doi: 10.1128/mSystems.00096-17 (PMC5698493; doi:10.1128/mSystems.00096-17)
Supplement: TABLE S1 [file sys006172154st9.pdf]

Table S1 Comparison of repeat structure in symbionts and free-living bacteria

Strain	Genome length (bp)	Repeat length (bp)	Repeat length (%)	Life-style*	No. Repeat loci	Repeat loci N50	Longest repeat locus (bp)
Spiroplasma citri R8-A2	1640878	666844	40.64	F	212	6597	40155
Sodalis glossinidius morsitans	4292502	580517	13.52	F	400	2814	14279
"*Candidatus* Didemnitutus mandela"	2684137	513494	19.13	T	5		108264
"*Candidatus* Hamiltonella defensa 5AT"	2169363	403291	18.59	T	277	2743	21540
Wolbachia wCle	1250060	222137	17.77	T	272	889	6484
Wolbachia wMel	1267782	182530	14.40	F	228	1261	4933
"*Candidatus* Tremblaya princeps PCIT"	138927	11893	8.56	O	6	5701	5701
Spiroplasma chrysopicola DF-1	1123322	88023	7.84	F	85	1321	4997
Streptomyces coelicolor A3_2	9054847	530189	5.86	N	429	2530	76766
Sorangium cellulosum So ce 56	13033779	741512	5.69	N	896	1493	24734
Pseudomonas fluorescens F113	6845832	285418	4.17	N	728	1247	5824
Spiroplasma sabaudiense Ar-1343	1075953	41544	3.86	F	48	1928	5064
Escherichia coli K-12 MG1655	4641652	171560	3.70	N	241	1335	5725
Spiroplasma taiwanense	1086278	27895	2.57	F	51	895	1782
Spiroplasma apis B31	1160554	26192	2.26	F	26	1363	2263
Spiroplasma syrphidicola EA-1	1107344	23847	2.15	F	46	777	1428
Spiroplasma culicicola AES-1	1175131	24914	2.12	F	50	849	2176
Bacillus subtilis 168	4215606	88834	2.11	N	76	5154	10834
Opitutus sp. GAS368	4150883	79732	1.92	N	242	417	1423
Spiroplasma eriocheiris DSM21848	1365714	24693	1.81	F	34	1130	1797
Serratia marcescens Db11	5113802	85838	1.68	N	108	2026	5722
"*Candidatus* Moranella endobia PCIT"	538294	8684	1.61	O	8	4022	4022
Spiroplasma atrichopogonis GNAT3597	1160484	17128	1.48	F	37	1222	2675
Pantoea vagans C9-1	4888338	71463	1.46	N	137	5269	5727
"*Candidatus* Pantoea carbekii"	1151074	15203	1.32	T	4	5114	5116
Sodalis praecaptivus HS1	5159425	63922	1.24	N	96	3901	6446

Spiroplasma diminutum CUAS-1	945296	11215	1.19	F	26	777	967
Buchnera aphidicola APS	655725	7354	1.12	O	2	7257	7257
"*Candidatus* Nasuia deltocephalinicola NAS-ALF"	112091	370	0.33	O	2	296	296
Serratia symbiotica SCc	1762765	3078	0.17	T	4	1449	1468
"*Candidatus* Sulcia muelleri PSPU"	285352	697	0.24	O	3	233	240
"*Candidatus* Hodgkinia cicadicola Dsem"	143795	130	0.09	O	1	130	130
Arsenophonus sp. CB	836724	78	0.01	T	1	78	78
"*Candidatus* Endolissoclinum faulkneri L2"	1481191	77	0.01	O	1	77	77

*Abbreviations: N, non-host-restricted; F, facultative; T, transitional; O, obligate
