# Supplementary material for: Increased Biosynthetic Gene Dosage in a Genome-Reduced Defensive Bacterial Symbiont
Source: mSystems. 2017 Nov 21;2(6):e00096-17. doi: 10.1128/mSystems.00096-17 (PMC5698493; doi:10.1128/mSystems.00096-17)
Supplement: TABLE S2 [file sys006172154st10.pdf]

Table S2 Putative pseudogenes with functional annotations in the "*Ca.* D. mandela" genome

Locus tag	Gene	EC_number	Product	Putative function
**CD822_00056**	yehT		Transcriptional regulatory protein YehT	regulation
**CD822_00098**	kdsD	5.3.1.13	Arabinose 5-phosphate isomerase KdsD	LPS biosynthesis
**CD822_00099**	fumA	4.2.1.2	Fumarate hydratase class I, aerobic	TCA cycle
**CD822_00122**	araD	5.1.3.4	L-ribulose-5-phosphate 4-epimerase	L-arabinose degradation
**CD822_00124**	araA	5.3.1.4	L-arabinose isomerase	L-arabinose degradation
**CD822_00250**	masK	2.7.10.2	Tyrosine-protein kinase MasK	
**CD822_00259**	ybeZ		PhoH-like protein	16S rRNA maturation?
**CD822_00309**	clpB_1		Chaperone protein ClpB	protein folding
**CD822_00310**	clpB_2		Chaperone protein ClpB	protein folding
**CD822_00353**	ribB	4.1.99.12	3,4-dihydroxy-2-butanone 4-phosphate synthase	riboflavin biosynthesis
**CD822_00507**	glpK	2.7.1.30	Glycerol kinase	glycerol utilization in glycolysis
**CD822_00684**		4.1.1.38	PPi-type phosphoenolpyruvate carboxykinase	gluconeogenesis
**CD822_00685**	feoB		Ferrous iron transport protein B	iron uptake
**CD822_00762**	deoC	4.1.2.4	Deoxyribose-phosphate aldolase	nucleic acid catabolism
**CD822_00764**	aldA	1.2.1.3	Putative aldehyde dehydrogenase AldA	folate biosynthesis, fucose and rhamnose metabolism
**CD822_00766**	acoD	1.2.1.3	Acetaldehyde dehydrogenase 2	
**CD822_00833**	xylA	5.3.1.5	Xylose isomerase	Xylose catabolism
**CD822_00875**	yehU	2.7.13.3	Sensor histidine kinase YehU	regulation
**CD822_00876**	ypdA	2.7.13.3	Sensor histidine kinase YpdA	regulation
**CD822_00994**	kdsA_1	2.5.1.55	2-dehydro-3-deoxyphosphooctonate aldolase	LPS biosynthesis
**CD822_00995**	kdsA_2	2.5.1.55	2-dehydro-3-deoxyphosphooctonate aldolase	LPS biosynthesis
**CD822_01026**	dcp	3.4.15.5	Peptidyl-dipeptidase dcp	Utilization of N-blocked peptides as carbon sources
**CD822_01040**	queA	2.4.99.17	S-adenosylmethionine:tRNA ribosyltransferase-isomerase	Formation of queuosine anticodon loop in some tRNAs
**CD822_01042**	ilvD	4.2.1.9	Dihydroxy-acid dehydratase	Isoleucine and valine biosynthesis
**CD822_01045**	ilvB1	2.2.1.6	Acetolactate synthase large subunit IlvB1	Isoleucine and valine biosynthesis
**CD822_01047**	ilvC	1.1.1.86	Ketol-acid reductoisomerase	Isoleucine and valine biosynthesis
**CD822_01049**	leuA	2.3.3.13	2-isopropylmalate synthase	Leucine biosynthesis
**CD822_01050**	leuB	1.1.1.85	3-isopropylmalate dehydrogenase	Leucine biosynthesis

**CD822_01097**	luxQ	2.7.13.3	Autoinducer 2 sensor kinase/phosphatase LuxQ	AI-2 quorum sensing
**CD822_01128**	trpB	4.2.1.20	Tryptophan synthase beta chain	Trypotphan biosynthesis
**CD822_01149**	waaA	2.4.99.12	3-deoxy-D-manno-octulosonic acid transferase	LPS biosynthesis
**CD822_01254**	nifJ	1.2.7.-	Pyruvate-flavodoxin oxidoreductase	Anaerobic fermentation
**CD822_01259**	ndhB	1.6.5.11	NAD(P)H-quinone oxidoreductase subunit 2, chloroplastic	Respiratory chain
**CD822_01351**	galE1	5.1.3.2	UDP-glucose 4-epimerase	Galactose catabolism
**CD822_01384**	cysT		Sulfate transport system permease protein CysT	Sulfate import
**CD822_01387**	cysW		Sulfate transport system permease protein CysW	Sulfate import
**CD822_01420**	glgC_1	2.7.7.27	Glucose-1-phosphate adenylyltransferase	Glycogen biosynthesis
**CD822_01421**	glgC_2	2.7.7.27	Glucose-1-phosphate adenylyltransferase	Glycogen biosynthesis
**CD822_01422**	glgC_3	2.7.7.27	Glucose-1-phosphate adenylyltransferase	Glycogen biosynthesis
**CD822_01423**	glgC_4	2.7.7.27	Glucose-1-phosphate adenylyltransferase	Glycogen biosynthesis
**CD822_01574**	phrA	4.1.99.3	Deoxyribodipyrimidine photo-lyase	Glycogen biosynthesis
**CD822_01627**	lptD		LPS-assembly protein LptD	LPS biosynthesis
**CD822_01655**	ribD		Riboflavin biosynthesis protein RibD	Riboflavin biosynthesis
**CD822_01710**	hemL	5.4.3.8	Glutamate-1-semialdehyde 2,1-aminomutase	Porphyrin biosynthesis
**CD822_01724**	mazG	3.6.1.1	Nucleoside triphosphate pyrophosphohydrolase/ pyrophosphatase MazG	Regulation of starvation response
**CD822_01774**	hsrA		putative transport protein HsrA	Drug efflux?
**CD822_01857**	polX_1		DNA polymerase/3'-5' exonuclease PolX	DNA repair
**CD822_01858**	polX_2		DNA polymerase/3'-5' exonuclease PolX	DNA repair
**CD822_01873**	mpl	6.3.2.45	UDP-N-acetylmuramate--L-alanyl-gamma-D-glutamyl-meso-2,6-diaminoheptandioate ligase	Murein recycling for peptidoglycan
**CD822_01937**	rfaF	2.-.-.-	ADP-heptose--LPS heptosyltransferase 2	LPS biosynthesis
**CD822_01965**	pgk/ tpi_1		Bifunctional PGK/TIM	Glycolysis
**CD822_01966**	pgk/ tpi_2		Bifunctional PGK/TIM	Glycolysis
**CD822_02049**	mlaE		putative phospholipid ABC transporter permease protein MlaE	Phospholipid transport
**CD822_02053**	proB_1	2.7.2.11	Glutamate 5-kinase	Proline biosynthesis
**CD822_02054**	proB_2	2.7.2.11	Glutamate 5-kinase 1	Proline biosynthesis
**CD822_02226**	rplY		50S ribosomal protein L25	Ribosomal protein
**CD822_02349**	lpxK_1	2.7.1.130	Tetraacyldisaccharide 4'-kinase	Lipid A biosynthesis
**CD822_02350**	lpxK_2	2.7.1.130	Tetraacyldisaccharide 4'-kinase	Lipid A biosynthesis
**CD822_02363**	malP	2.4.1.1	Maltodextrin phosphorylase	Maltodextrin utilization
**CD822_02378**	xpsE		Type II secretion system protein E	Protein secretion

**CD822_02389**	acsA	6.2.1.1	Acetyl-coenzyme A synthetase	Acetyl-CoA biosynthesis from acetate
**CD822_02427**	arnB	2.6.1.87	UDP-4-amino-4-deoxy-L-arabinose--oxoglutarate aminotransferase	Lipid A modification
**CD822_02428**	desV	2.6.1.106	dTDP-3-amino-3,4,6-trideoxy-alpha-D-glucose transaminase	Desosamine biosynthesis
**CD822_02456**	gmd	4.2.1.47	GDP-mannose 4,6-dehydratase	LPS biosynthesis
**CD822_02458**	fcl_1	1.1.1.271	GDP-L-fucose synthase	LPS biosynthesis
**CD822_02494**	resA		Thiol-disulfide oxidoreductase ResA	Cytochrome c assembly
**CD822_02510**	fbpC2	3.6.3.30	Fe(3+) ions import ATP-binding protein FbpC 2	Iron uptake
**CD822_02565**	glgA	2.4.1.21	Glycogen synthase	Glycogen biosynthesis
**CD822_02629**	mfppA	3.1.3.79	Mannosylfructose-phosphate phosphatase	Osmotic stress response
**CD822_02652**	ettA		Energy-dependent translational throttle protein EttA	Regulation of translation
**CD822_02727**	aceB	2.3.3.9	Malate synthase	Glyoxylate cycle
**CD822_02780**	rfaQ	2.-.-.-	Lipopolysaccharide core heptosyltransferase RfaQ	LPS biosynthesis
**CD822_02795**	ptsI_1	2.7.3.9	Phosphoenolpyruvate-protein phosphotransferase	Sugar uptake
**CD822_02796**	ptsI_2	2.7.3.9	Phosphoenolpyruvate-protein phosphotransferase	Sugar uptake
**CD822_02839**		1.2.1.26	Alpha-ketoglutaric semialdehyde dehydrogenase	Xylose catabolism
**CD822_02843**	nsaE	4.1.2.45	Trans-O-hydroxybenzylidenepyruvate hydratase-aldolase	Naphthalene degradation
**CD822_02872**	tal	2.2.1.2	Transaldolase	Pentose phosphate pathway
**CD822_02889**	kdpA		Potassium-transporting ATPase potassium-binding subunit	Potassium transport
**CD822_02895**	kdsB_1	2.7.7.38	3-deoxy-manno-octulosonate cytidylyltransferase	LPS biosynthesis
**CD822_02896**	kdsB_2	2.7.7.38	3-deoxy-manno-octulosonate cytidylyltransferase	LPS biosynthesis
**CD822_02897**	crh		HPr-like protein Crh	Carbon catabolite repression
