# Supplementary figures and images for: Increased Biosynthetic Gene Dosage in a Genome-Reduced Defensive Bacterial Symbiont
Source: mSystems. 2017 Nov 21;2(6):e00096-17. doi: 10.1128/mSystems.00096-17 (PMC5698493; doi:10.1128/mSystems.00096-17)

A

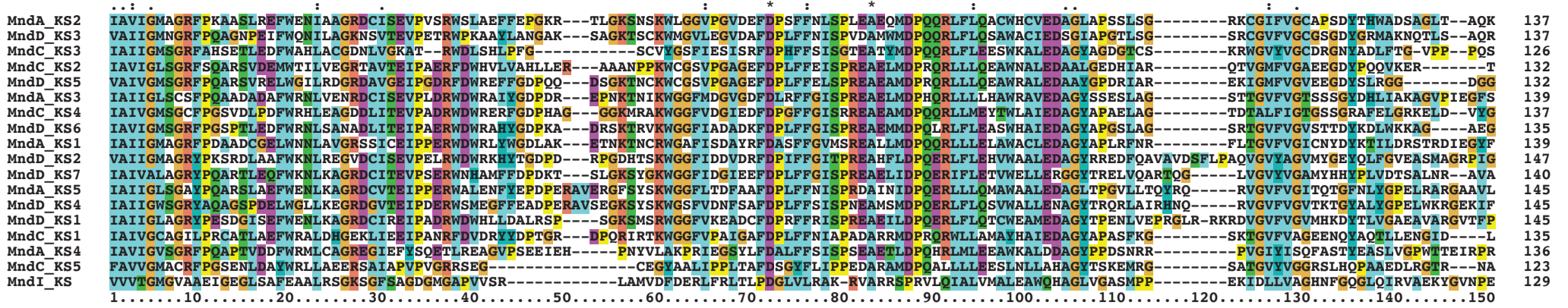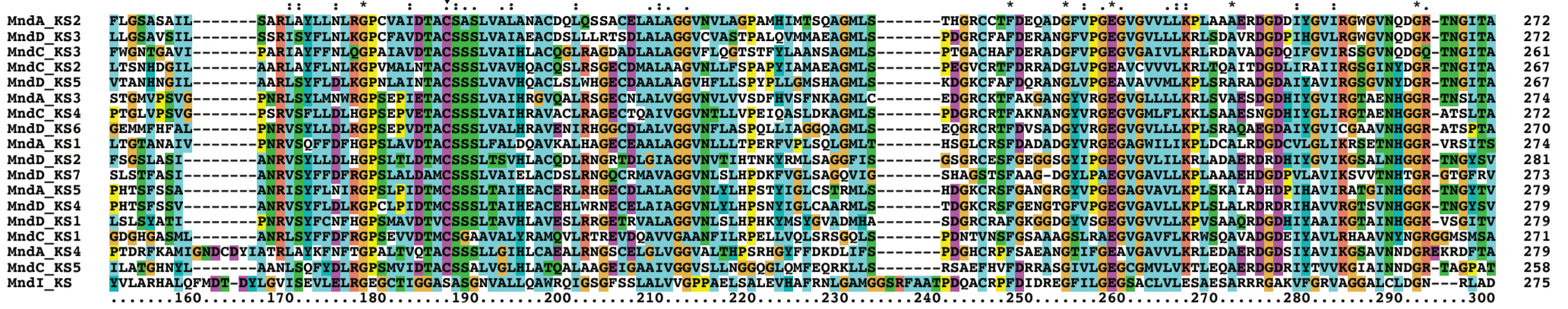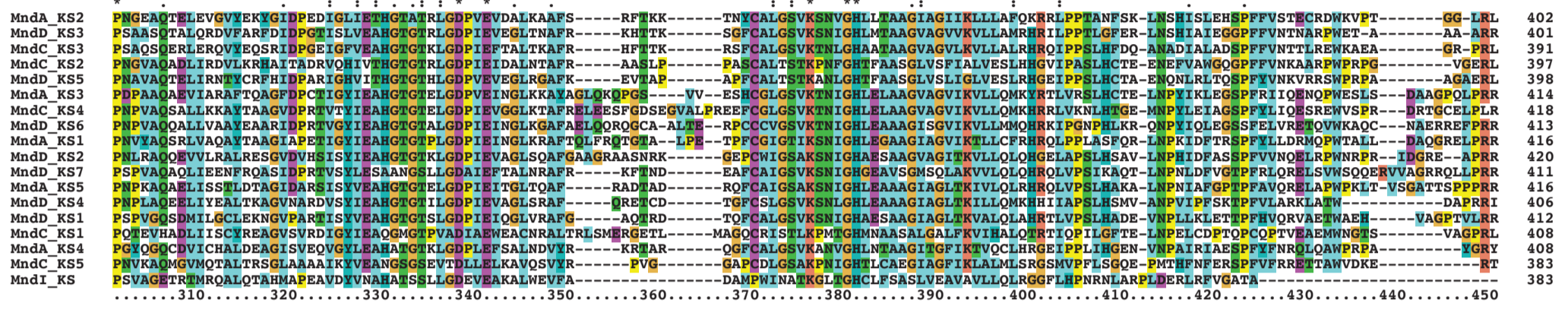

B

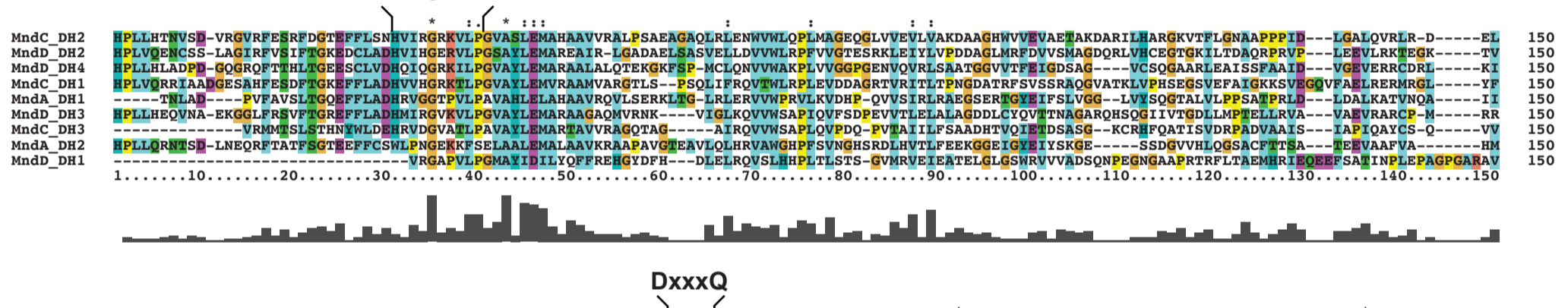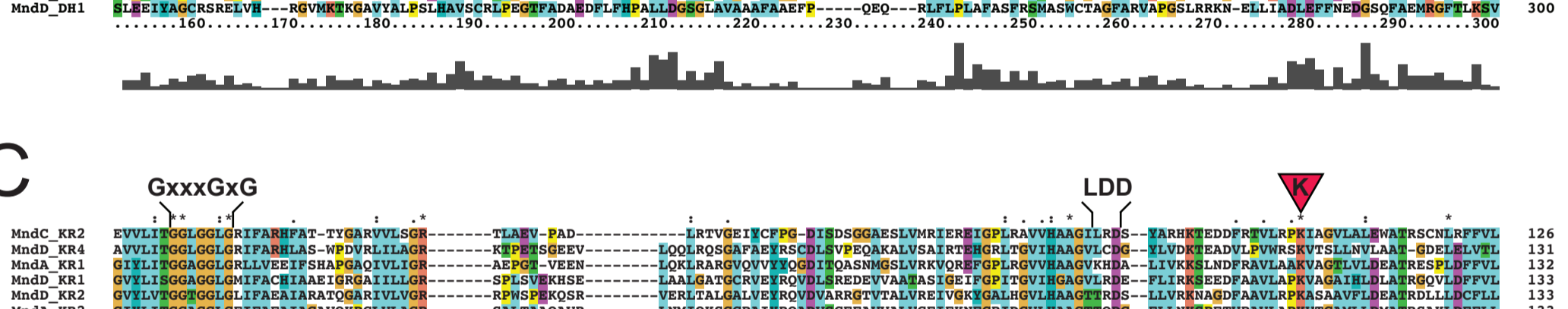

C

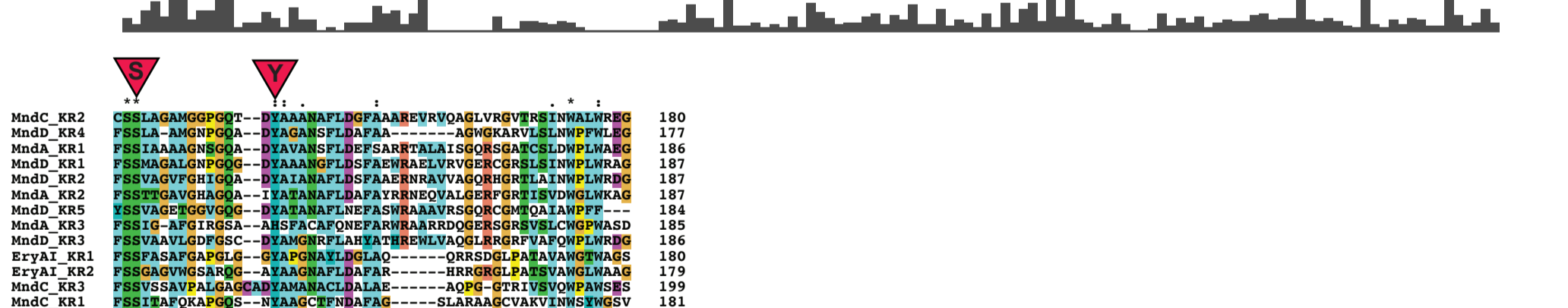

D

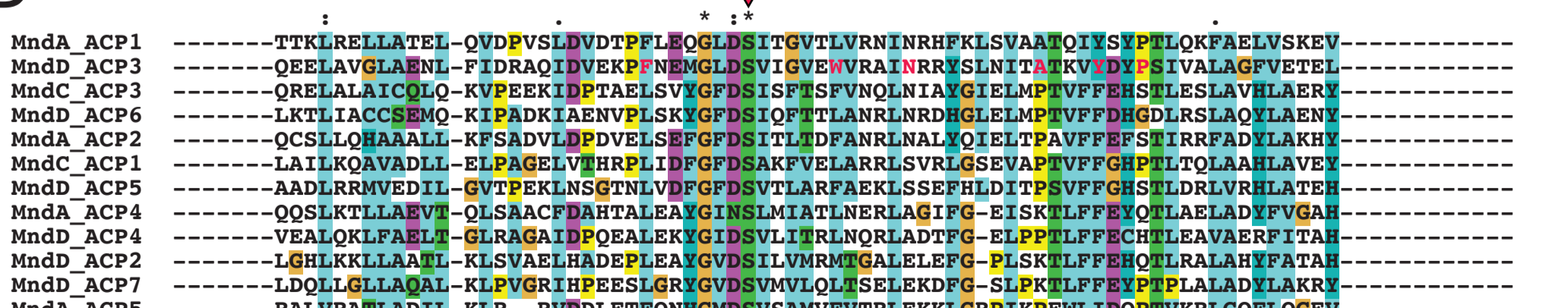

Supplement: FIG S5 [file sys006172154sf5.pdf]
