# Supplementary material for: Increased Biosynthetic Gene Dosage in a Genome-Reduced Defensive Bacterial Symbiont
Source: mSystems. 2017 Nov 21;2(6):e00096-17. doi: 10.1128/mSystems.00096-17 (PMC5698493; doi:10.1128/mSystems.00096-17)
Supplement: FIG S8 [file sys006172154sf8.pdf]

COX1 Amplicon 1

	Lissoclinum sp. TIC-2013-079	*Lissoclinum patella* L6 AHZ94369.1	*Lissoclinum patella* L5 AHZ94363.1	*Lissoclinum patella* L2 AHZ94350.1	*Lissoclinum punctatum* BAG71823.1	*Lissoclinum bistratum* BAI99553.1	*Lissoclinum timorense* BAI99558.1	*Lissoclinum timorense* BAI99557.1	*Lissoclinum bistratum* BAG71824.1	*Lissoclinum timorense* BAI99556.1	*Lissoclinum bistratum* BAI99555.1	*Lissoclinum bistratum* BAI99554.1	*Lissoclinum patella* BAK32991.1
Lissoclinum sp. TIC-2013-079	100	73	73	72.4	82.2	77.7	78	77.8	77.4	76.7	76.7	77.2	75.7
Lissoclinum patella L6 AHZ94369.1	73	100	100	85.9	77.7	83.2	83.4	83.5	83.5	82.9	82.9	82.9	87.2
Lissoclinum patella L5 AHZ94363.1	73	100	100	85.9	77.7	83.2	83.4	83.5	83.5	82.9	82.9	82.9	87.2
Lissoclinum patella L2 AHZ94350.1	72.4	85.9	85.9	100	75.7	82.7	82.9	82.8	82.7	81.7	81.7	81.7	85.5
Lissoclinum punctatum BAG71823.1	82.2	77.7	77.7	75.7	100	78.2	78.4	78.5	78.2	75.7	75.7	75.7	76.2
Lissoclinum bistratum BAI99553.1	77.7	83.2	83.2	82.7	78.2	100	99.8	99.8	99.5	83.9	83.9	83.9	81.5
Lissoclinum timorense BAI99558.1	78	83.4	83.4	82.9	78.4	99.8	100	100	99.7	84.2	84.2	84.2	81.7
Lissoclinum timorense BAI99557.1	77.8	83.5	83.5	82.8	78.5	99.8	100	100	99.7	84.3	84.3	84	81.8
Lissoclinum bistratum BAG71824.1	77.4	83.5	83.5	82.7	78.2	99.5	99.7	99.7	100	83.7	83.7	83.7	82
Lissoclinum timorense BAI99556.1	76.7	82.9	82.9	81.7	75.7	83.9	84.2	84.3	83.7	100	100	99.5	82.5
Lissoclinum bistratum BAI99555.1	76.7	82.9	82.9	81.7	75.7	83.9	84.2	84.3	83.7	100	100	99.5	82.5
Lissoclinum bistratum BAI99554.1	77.2	82.9	82.9	81.7	75.7	83.9	84.2	84	83.7	99.5	99.5	100	82.5
Lissoclinum patella BAK32991.1	75.7	87.2	87.2	85.5	76.2	81.5	81.7	81.8	82	82.5	82.5	82.5	100

COX1 Amplicon 2

	Lissoclinum sp. TIC-2013-079	*Lissoclinum patella* L6 AHZ94369.1	*Lissoclinum patella* L5 AHZ94363.1	*Lissoclinum patella* L2 AHZ94350.1	*Lissoclinum patella* 07-110 AHZ18353.1	*Lissoclinum patella* 07-005 AHZ18352.1	*Lissoclinum patella* 07-002B AHZ18351.1	*Lissoclinum patella* 03-005 AHZ18350.1	*Lissoclinum patella* 07-103 AHZ18349.1	*Lissoclinum patella* L4 AHZ18348.1	*Lissoclinum patella* E11-097 AHZ18347.1	*Lissoclinum patella* 05-044 AHZ18346.1	*Lissoclinum patella* 05-027 AHZ18345.1	*Lissoclinum patella* 05-039 AHZ18344.1	*Lissoclinum patella* 05-033 AHZ18343.1	*Lissoclinum patella* L3 AHZ18342.1	*Lissoclinum verrilli* AGF29554.1
Lissoclinum sp. TIC-2013-079	100	73	73	72.4	72.5	73.3	72.6	72.8	73.7	74.7	71.9	74.5	74.4	74.4	74.5	73.5	75.3
Lissoclinum patella L6 AHZ94369.1	73	100	100	85.9	81.1	81	80.7	81	81.6	82.4	81.5	83.5	83.1	83.1	83.1	88	67.5
Lissoclinum patella L5 AHZ94363.1	73	100	100	85.9	81.1	81	80.7	81	81.6	82.4	81.5	83.5	83.1	83.1	83.1	88	67.5
Lissoclinum patella L2 AHZ94350.1	72.4	85.9	85.9	100	81.5	81.8	81.1	81.3	82.1	82.5	80.9	82.7	82.8	82.8	82.4	86.4	66.5
Lissoclinum patella 07-110 AHZ18353.1	72.5	81.1	81.1	81.5	100	98.1	98	98.1	97.9	97.8	88.2	90.1	90.4	90.4	90.1	81.8	65.4
Lissoclinum patella 07-005 AHZ18352.1	73.3	81	81	81.8	98.1	100	99.8	99.6	98.5	98.5	88.6	90.5	91	90.8	90.8	82.4	65.6
Lissoclinum patella 07-002B AHZ18351.1	72.6	80.7	80.7	81.1	98	99.8	100	99.4	98.3	98.4	88.5	90.2	90.8	90.5	90.5	82.3	65.6
Lissoclinum patella 03-005 AHZ18350.1	72.8	81	81	81.3	98.1	99.6	99.4	100	98.4	98.3	88.6	90.4	90.9	90.7	90.6	82.4	65.8
Lissoclinum patella 07-103 AHZ18349.1	73.7	81.6	81.6	82.1	97.9	98.5	98.3	98.4	100	100	88.9	91.2	91.3	91.3	91.2	82.4	66.5
Lissoclinum patella L4 AHZ18348.1	74.7	82.4	82.4	82.5	97.8	98.5	98.4	98.3	100	100	88.9	91.5	91.5	91.5	91.5	82.4	68.6
Lissoclinum patella E11-097 AHZ18347.1	71.9	81.5	81.5	80.9	88.2	88.6	88.5	88.6	88.9	88.9	100	95.3	95.6	95.3	95.1	80	67.5
Lissoclinum patella 05-044 AHZ18346.1	74.5	83.5	83.5	82.7	90.1	90.5	90.2	90.4	91.2	91.5	95.3	100	99.8	99.8	99.4	82	66.4
Lissoclinum patella 05-027 AHZ18345.1	74.4	83.1	83.1	82.8	90.4	91	90.8	90.9	91.3	91.5	95.6	99.8	100	99.8	99.4	82.3	67.1
Lissoclinum patella 05-039 AHZ18344.1	74.4	83.1	83.1	82.8	90.4	90.8	90.5	90.7	91.3	91.5	95.3	99.8	99.8	100	99.4	82.3	66.9
Lissoclinum patella 05-033 AHZ18343.1	74.5	83.1	83.1	82.4	90.1	90.8	90.5	90.6	91.2	91.5	95.1	99.4	99.4	99.4	100	82.4	67
Lissoclinum patella L3 AHZ18342.1	73.5	88	88	86.4	81.8	82.4	82.3	82.4	82.4	82.4	80	82	82.3	82.3	82.4	100	69
Lissoclinum verrilli AGF29554.1	75.3	67.5	67.5	66.5	65.4	65.6	65.6	65.8	66.5	68.6	67.5	66.4	67.1	66.9	67	69	100
