# Supplementary material for: Increased Biosynthetic Gene Dosage in a Genome-Reduced Defensive Bacterial Symbiont
Source: mSystems. 2017 Nov 21;2(6):e00096-17. doi: 10.1128/mSystems.00096-17 (PMC5698493; doi:10.1128/mSystems.00096-17)
Supplement: FIG S4 [file sys006172154sf4.pdf]

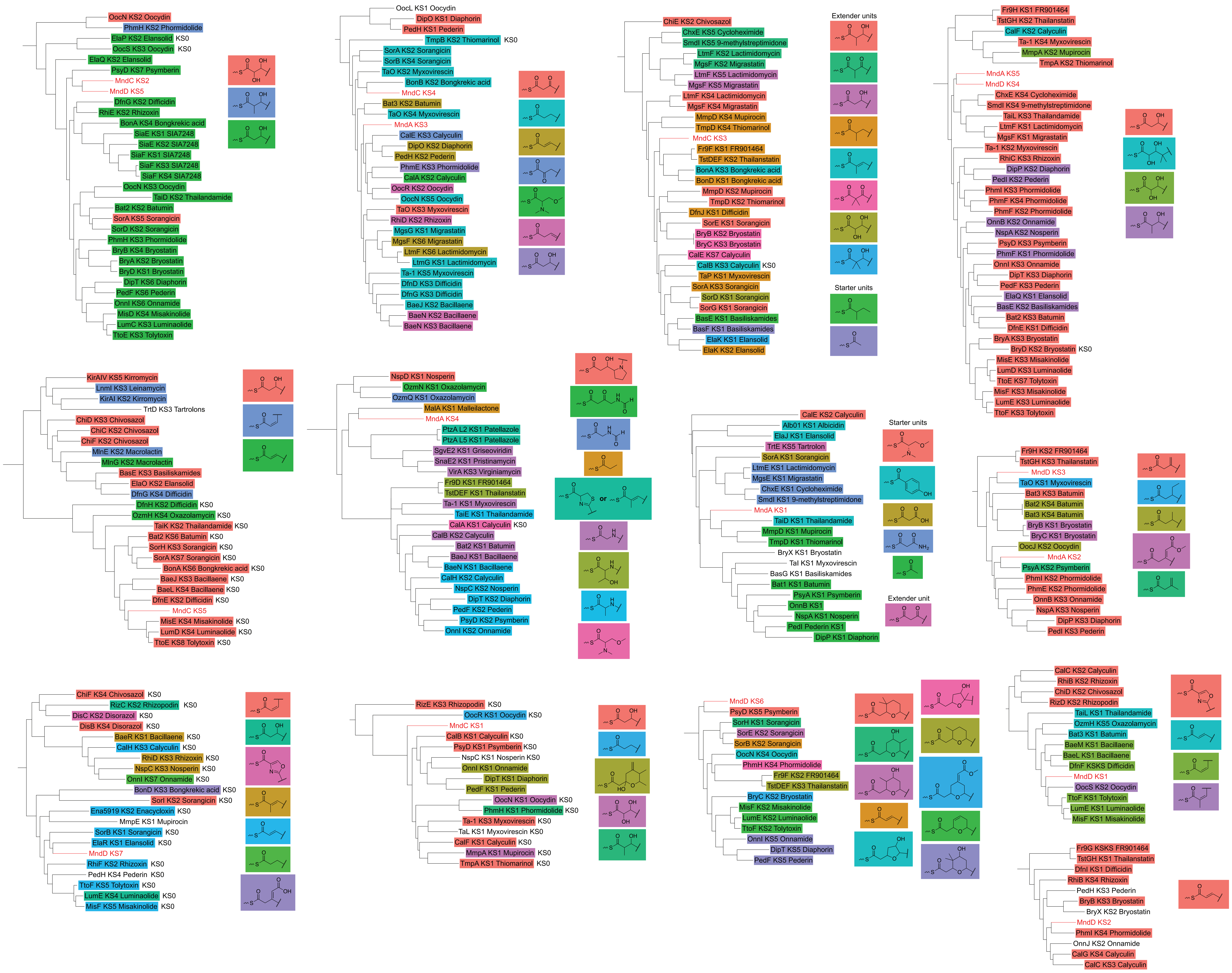
OocN KS2 Oocydin
PhmH KS2 Phormidolide
ElaP KS2 Elansolid KS0
OocS KS3 Oocydin KS0
ElaQ KS2 Elansolid
PsyD KS7 Psymberin
MndC KS2
MndD KS5
DfnG KS2 Difficidin
RhiE KS2 Rhizoxin
BonA KS4 Bongkrekic acid
SiaE KS1 SIA7248
SiaE KS2 SIA7248
SiaF KS1 SIA7248
SiaF KS3 SIA7248
SiaF KS4 SIA7248
OocN KS3 Oocydin
TaiD KS2 Thailandamide
Bat2 KS2 Batumin
SorA KS5 Sorangicin
SorD KS2 Sorangicin
PhmH KS3 Phormidolide
BryB KS4 Bryostatin
BryA KS2 Bryostatin
BryD KS1 Bryostatin
DipT KS6 Diaphorin
PedF KS6 Pederin
OnnI KS6 Onnamide
MisD KS4 Misakinolide
LumC KS3 Luminaolide
TtoE KS3 Tolytoxin
OocL KS1 Oocydin
DipO KS1 Diaphorin
PedH KS1 Pederin
TmpB KS2 Thiomarinol KS0
SorA KS2 Sorangicin
SorB KS4 Sorangicin
TaO KS2 Myxovirescin
BonB KS2 Bongkrekic acid
MndC KS4
Bat3 KS2 Batumin
TaO KS4 Myxovirescin
MndA KS3
CalE KS3 Calyculin
DipO KS2 Diaphorin
PedH KS2 Pederin
PhmE KS3 Phormidolide
CalA KS2 Calyculin
OocR KS2 Oocydin
OocN KS5 Oocydin
TaO KS3 Myxovirescin
RhiD KS2 Rhizoxin
MgsG KS1 Migrastatin
MgsF KS6 Migrastatin
LtmF KS6 Lactimidomycin
LtmG KS1 Lactimidomycin
Ta-1 KS5 Myxovirescin
DfnD KS3 Difficidin
DfnG KS3 Difficidin
BaeJ KS2 Bacillaene
BaeN KS2 Bacillaene
BaeN KS3 Bacillaene
ChiE KS2 Chivosazol
ChxE KS5 Cycloheximide
SmdI KS5 9-methylstreptimidone
LtmF KS2 Lactimidomycin
MgsF KS2 Migrastatin
LtmF KS5 Lactimidomycin
MgsF KS5 Migrastatin
LtmF KS4 Lactimidomycin
MgsF KS4 Migrastatin
MmpD KS4 Mupirocin
TmpD KS4 Thiomarinol
MndC KS3
Fr9F KS1 FR901464
TstDEF KS2 Thailanstatin
BonA KS3 Bongkrekic acid
BonD KS1 Bongkrekic acid
MmpD KS2 Mupirocin
TmpD KS2 Thiomarinol
DfnJ KS1 Difficidin
SorE KS1 Sorangicin
BryB KS2 Bryostatin
BryC KS3 Bryostatin
CalE KS7 Calyculin
CalB KS3 Calyculin KS0
TaP KS1 Myxovirescin
SorA KS3 Sorangicin
SorD KS1 Sorangicin
SorG KS1 Sorangicin
BasE KS1 Basiliskamides
BasF KS1 Basiliskamides
ElaK KS1 Elansolid
ElaK KS2 Elansolid
Extender units
Starter units
Fr9H KS1 FR901464
TstGH KS2 Thailanstatin
CalF KS2 Calyculin
Ta-1 KS4 Myxovirescin
MmpA KS2 Mupirocin
TmpA KS2 Thiomarinol
MndA KS5
MndD KS4
ChxE KS4 Cycloheximide
SmdI KS4 9-methylstreptimidone
TaiL KS3 Thailandamide
LtmF KS1 Lactimidomycin
MgsF KS1 Migrastatin
Ta-1 KS2 Myxovirescin
RhiC KS3 Rhizoxin
DipP KS2 Diaphorin
PedI KS2 Pederin
PhmI KS3 Phormidolide
PhmF KS4 Phormidolide
PhmF KS2 Phormidolide
OnnB KS2 Onnamide
NspA KS2 Nosperin
PsyD KS3 Psymberin
PhmF KS1 Phormidolide
OnnI KS3 Onnamide
DipT KS3 Diaphorin
PedF KS3 Pederin
ElaQ KS1 Elansolid
BasE KS2 Basiliskamides
Bat2 KS3 Batumin
DfnE KS1 Difficidin
BryA KS3 Bryostatin
BryD KS2 Bryostatin KS0
MisE KS3 Misakinolide
LumD KS3 Luminaolide
TtoE KS7 Tolytoxin
MisF KS3 Misakinolide
LumE KS3 Luminaolide
TtoF KS3 Tolytoxin
KirAIV KS5 Kirromycin
LnmI KS3 Leinamycin
KirAI KS2 Kirromycin
TrtD KS3 Tartrolons
ChiD KS3 Chivosazol
ChiC KS2 Chivosazol
ChiF KS2 Chivosazol
MlnE KS2 Macrolactin
MlnG KS2 Macrolactin
BasE KS3 Basiliskamides
ElaO KS2 Elansolid
DfnG KS4 Difficidin
DfnH KS2 Difficidin KS0
OzmH KS4 Oxazolamycin KS0
TaiK KS2 Thailandamide KS0
Bat2 KS6 Batumin KS0
SorH KS3 Sorangicin KS0
SorA KS7 Sorangicin KS0
BonA KS6 Bongkrekic acid KS0
BaeJ KS3 Bacillaene KS0
BaeL KS4 Bacillaene KS0
DfnE KS2 Difficidin KS0
MndC KS5
MisE KS4 Misakinolide KS0
LumD KS4 Luminaolide KS0
TtoE KS8 Tolytoxin KS0
NspD KS1 Nosperin
OzmN KS1 Oxazolamycin
OzmQ KS1 Oxazolamycin
MalA KS1 Malleilactone
MndA KS4
PtzA L2 KS1 Patellazole
PtzA L5 KS1 Patellazole
SgvE2 KS1 Griseoviridin
SnaE2 KS1 Pristinamycin
VirA KS3 Virginiamycin
Fr9D KS1 FR901464
TstDEF KS1 Thailanstatin
Ta-1 KS1 Myxovirescin
TaiE KS1 Thailandamide
CalA KS1 Calyculin KS0
CalB KS2 Calyculin
Bat2 KS1 Batumin
BaeJ KS1 Bacillaene
BaeN KS1 Bacillaene
CalH KS2 Calyculin
NspC KS2 Nosperin
DipT KS2 Diaphorin
PedF KS2 Pederin
PsyD KS2 Psymberin
OnnI KS2 Onnamide
or
CalE KS2 Calyculin
Alb01 KS1 Albicidin
ElaJ KS1 Elansolid
TrtE KS5 Tartrolon
SorA KS1 Sorangicin
LtmE KS1 Lactimidomycin
MgsE KS1 Migrastatin
ChxE KS1 Cycloheximide
SmdI KS1 9-methylstreptimidone
MndA KS1
TaiD KS1 Thailandamide
MmpD KS1 Mupirocin
TmpD KS1 Thiomarinol
BryX KS1 Bryostatin
TaI KS1 Myxovirescin
BasG KS1 Basiliskamides
Bat1 KS1 Batumin
PsyA KS1 Psymberin
OnnB KS1
NspA KS1 Nosperin
PedI Pederin KS1
DipP KS1 Diaphorin
Starter units
Extender unit
Fr9H KS2 FR901464
TstGH KS3 Thailanstatin
MndD KS3
TaO KS1 Myxovirescin
Bat3 KS3 Batumin
Bat2 KS4 Batumin
Bat3 KS4 Batumin
BryB KS1 Bryostatin
BryC KS1 Bryostatin
OocJ KS2 Oocydin
MndA KS2
PsyA KS2 Psymberin
PhmI KS2 Phormidolide
PhmE KS2 Phormidolide
OnnB KS3 Onnamide
NspA KS3 Nosperin
DipP KS3 Diaphorin
PedI KS3 Pederin
ChiF KS4 Chivosazol KS0
RizC KS2 Rhizopodin KS0
DisC KS2 Disorazol KS0
DisB KS4 Disorazol KS0
BaeR KS1 Bacillaene KS0
CalH KS3 Calyculin KS0
RhiD KS3 Rhizoxin KS0
NspC KS3 Nosperin KS0
OnnI KS7 Onnamide KS0
BonD KS3 Bongkrekic acid KS0
SorI KS2 Sorangicin KS0
Ena5919 KS2 Enacyloxin KS0
MmpE KS1 Mupirocin
SorB KS1 Sorangicin KS0
ElaR KS1 Elansolid KS0
MndD KS7
RhiF KS2 Rhizoxin KS0
PedH KS4 Pederin KS0
TtoF KS5 Tolytoxin KS0
LumE KS4 Luminaolide KS0
MisF KS5 Misakinolide KS0
RizE KS3 Rhizopodin KS0
OocR KS1 Oocydin KS0
MndC KS1
CalB KS1 Calyculin KS0
PsyD KS1 Psymberin KS0
NspC KS1 Nosperin KS0
OnnI KS1 Onnamide
DipT KS1 Diaphorin
PedF KS1 Pederin
OocN KS1 Oocydin KS0
PhmH KS1 Phormidolide KS0
Ta-1 KS3 Myxovirescin KS0
TaL KS1 Myxovirescin KS0
CalF KS1 Calyculin KS0
MmpA KS1 Mupirocin KS0
TmpA KS1 Thiomarinol KS0
MndD KS6
PsyD KS5 Psymberin
SorH KS1 Sorangicin
SorE KS2 Sorangicin
SorB KS2 Sorangicin
OocN KS4 Oocydin
PhmH KS4 Phormidolide
Fr9F KS2 FR901464
TstDEF KS3 Thailanstatin
BryC KS2 Bryostatin
MisF KS2 Misakinolide
LumE KS2 Luminaolide
TtoF KS2 Tolytoxin
OnnI KS5 Onnamide
DipT KS5 Diaphorin
PedF KS5 Pederin
CalC KS2 Calyculin
RhiB KS2 Rhizoxin
ChiD KS2 Chivosazol
RizD KS2 Rhizopodin
TaiL KS1 Thailandamide
OzmH KS5 Oxazolamycin
Bat3 KS1 Batumin
BaeM KS1 Bacillaene
BaeL KS1 Bacillaene
DfnF KSKS Difficidin
MndD KS1
OocS KS2 Oocydin
TtoF KS1 Tolytoxin
LumE KS1 Luminaolide
MisF KS1 Misakinolide
Fr9G KSKS FR901464
TstGH KS1 Thailanstatin
DfnI KS1 Difficidin
RhiB KS4 Rhizoxin
PedH KS3 Pederin
BryB KS3 Bryostatin
BryX KS2 Bryostatin
MndD KS2
PhmI KS4 Phormidolide
OnnJ KS2 Onnamide
CalG KS4 Calyculin
CalC KS3 Calyculin
